# Supplementary figures and images for: Ligand dependent gene regulation by transient ERα clustered enhancers
Source: PLoS Genet. 2020 Jan 6;16(1):e1008516. doi: 10.1371/journal.pgen.1008516 (PMC6975561; doi:10.1371/journal.pgen.1008516)

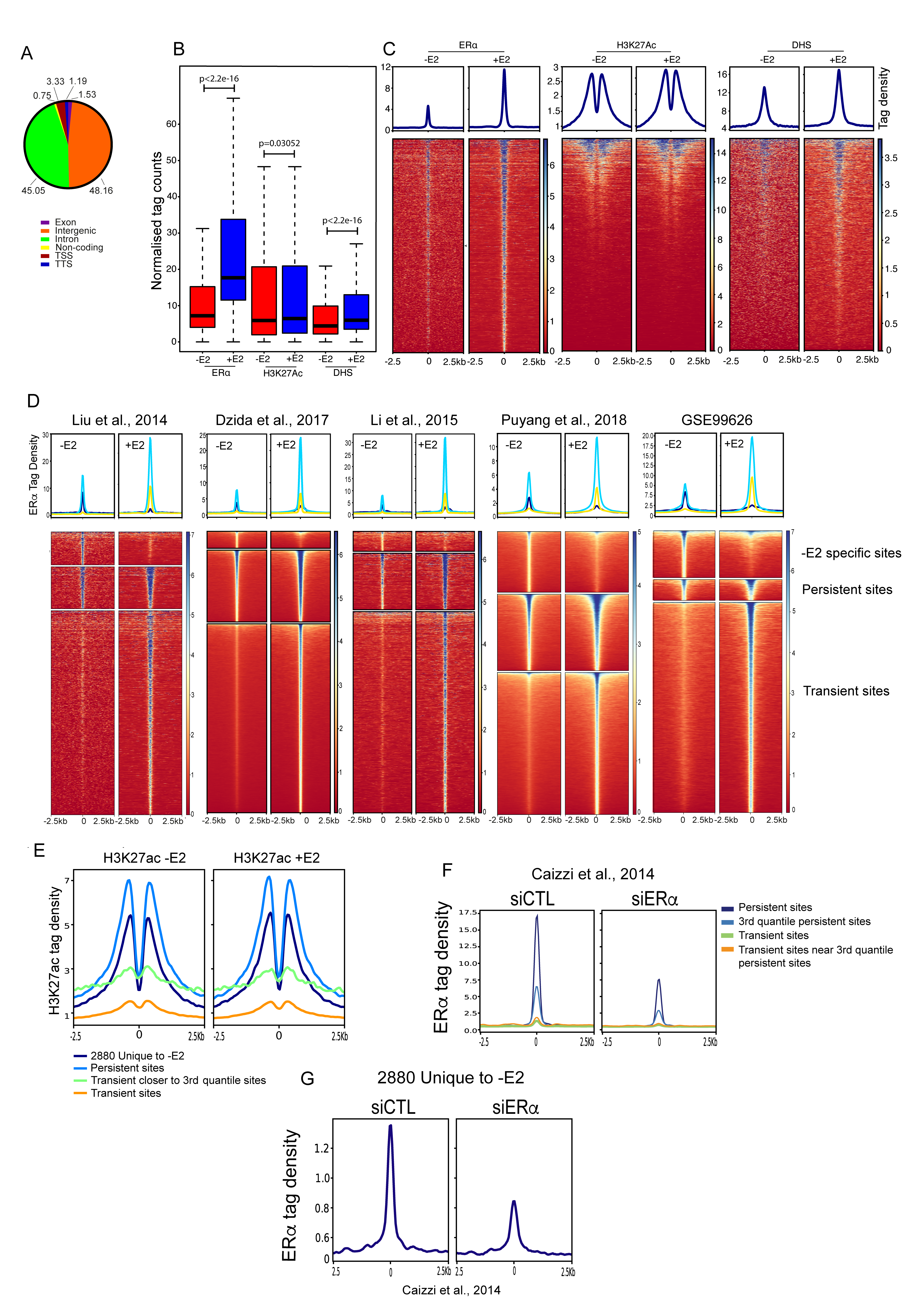

Supplement: S1 Fig — (A) Venn diagram shows distribution of ERα on various genomic regions. (B) Strength of ERα occupancy, DHS, and H3K27ac levels at ERα peaks before and after E2 treatments. (C) Heatmaps of ERα, DHS, and H3K27ac 2.5 kb upstream and downstream regions from the center of the ERα peaks in untreated and E2 treated condition. (D) Heatmaps depicting the relative binding of ERα on -E2 specific unique sites, persistent sites and transient sites across different datasets. (E) H3K27ac enrichment plot in minus and plus E2 on 2880 -E2 unique, persistent, transient closer to 3rd quantile, and transient sites. (F) ERα binding strength is affected upon siRNA mediated knockdown of ERα in untreated cells (data from Caizzi et al., 2014.) (G) ERα binding strength on 2880 -E2 unique sites is affected upon siRNA mediated knockdown of ERα in untreated cells (data from Caizzi et al., 2014). (TIF) [file pgen.1008516.s001.tif]

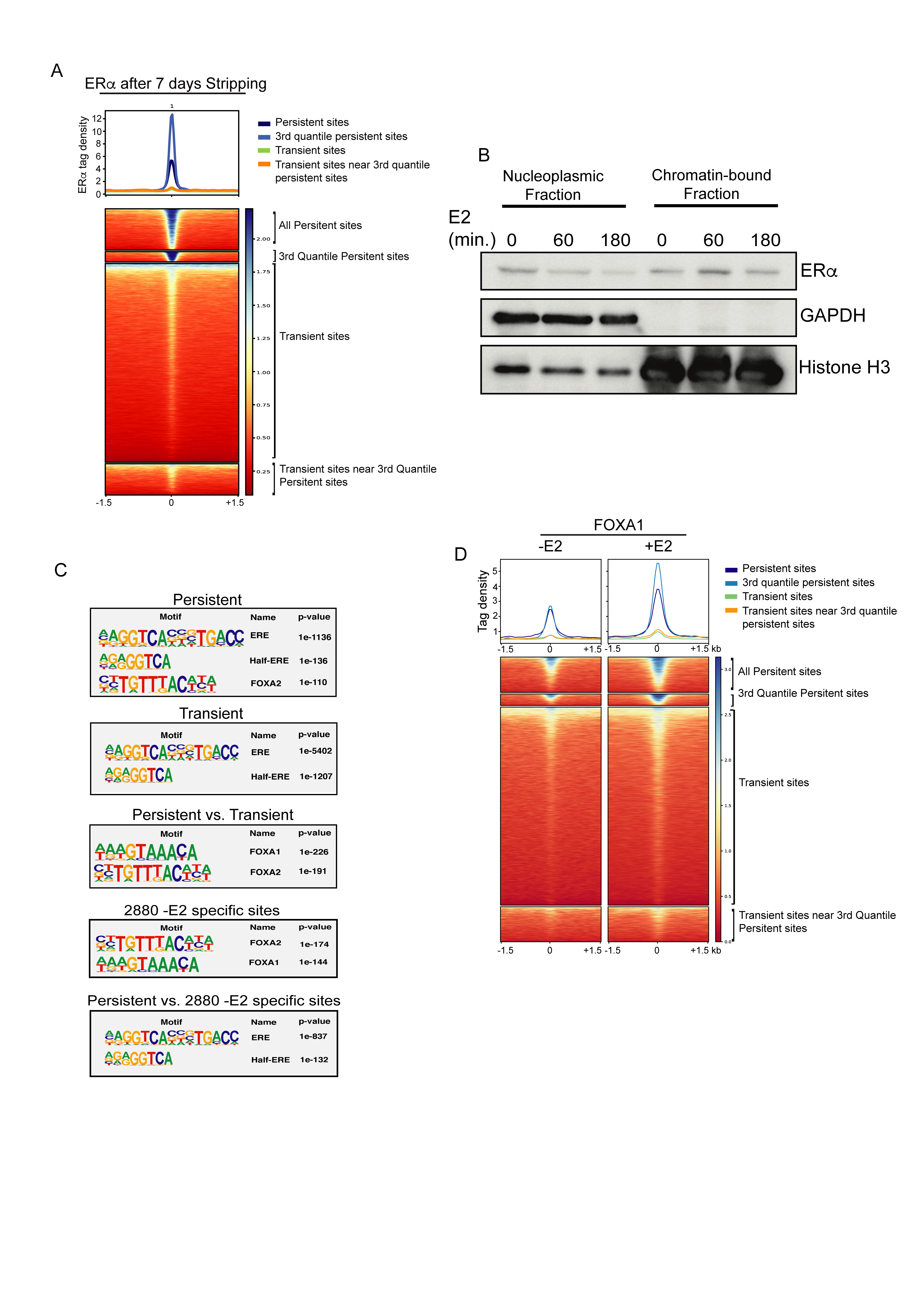

Supplement: S2 Fig — (A) Heatmap showing the relative ERα binding strength on various classes of ERα binding sites after 7 days of stripping and frequent change of media. (B) Immunoblot for ERα, GAPDH and Histone H3 in nucleoplasmic (soluble) and chromatin bound biochemical fractions in cells stripped for 7 days followed by 60 and 180 min E2 treatments. (C) (top panel) Known Motif enrichment analysis identifies full ERE in both persistent (p = 10−1136) and transient sites (p = 10−5402) whereas FOXA1 is enriched uniquely in persistent with p = 10−226 and in -E2 unique sites p = 10−174. (D) Heatmaps representing the strength of FOXA1 binding in different categories of ERα peaks in treated and untreated cells. Strength was measured at 1.5 kb upstream and downstream of center of ERα peak. (TIF) [file pgen.1008516.s002.tif]

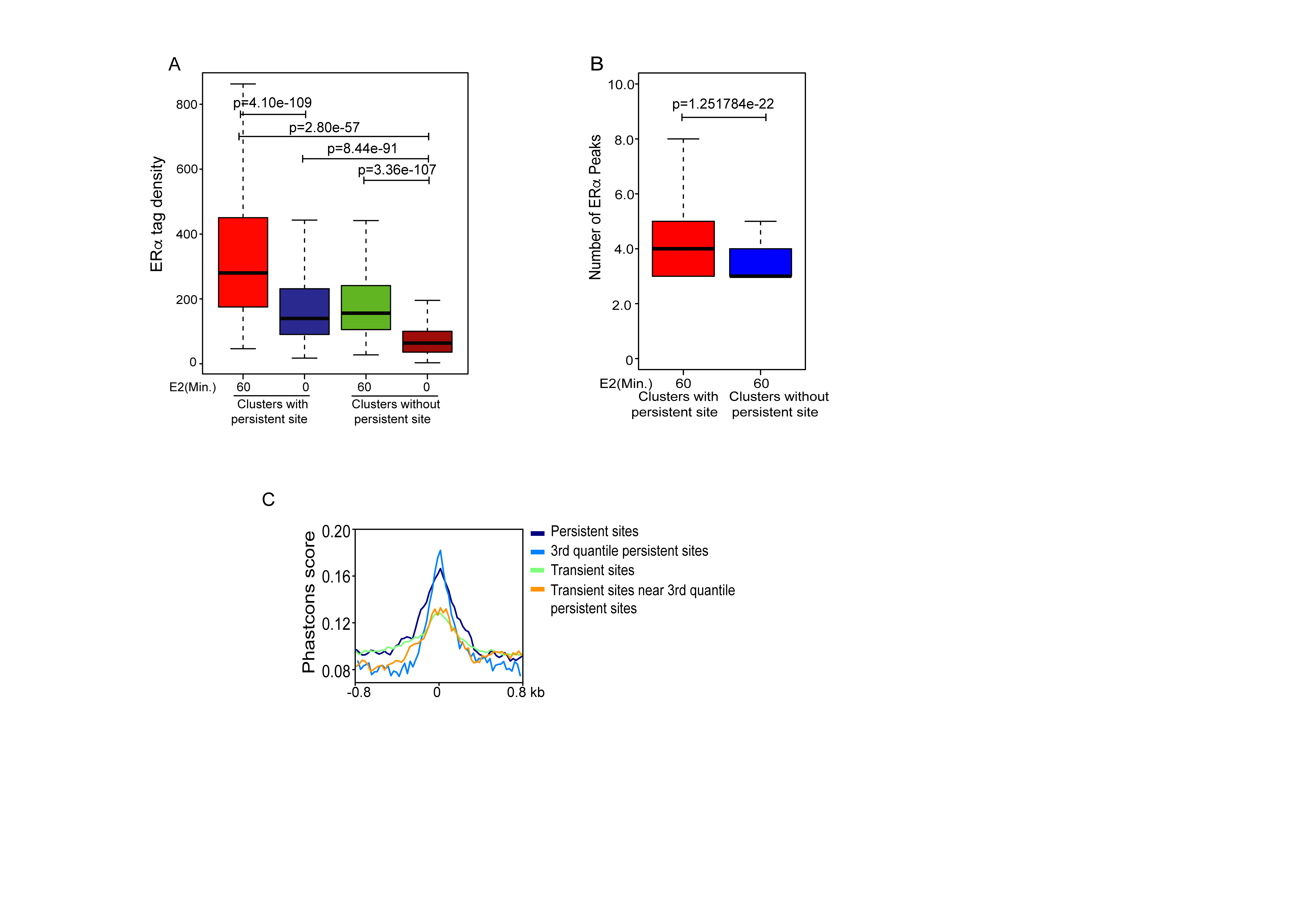

Supplement: S3 Fig — (A) ERα binding strength in clusters with and without persistent sites in E2 untreated and treated conditions. (B) Number of ERα peaks are greater in clusters with persistent site as compared to clusters without persistent sites. (C) Phast-cons score of persistent, 3rd quantile persistent, transient, and transient near persistent sites. (TIF) [file pgen.1008516.s003.tif]

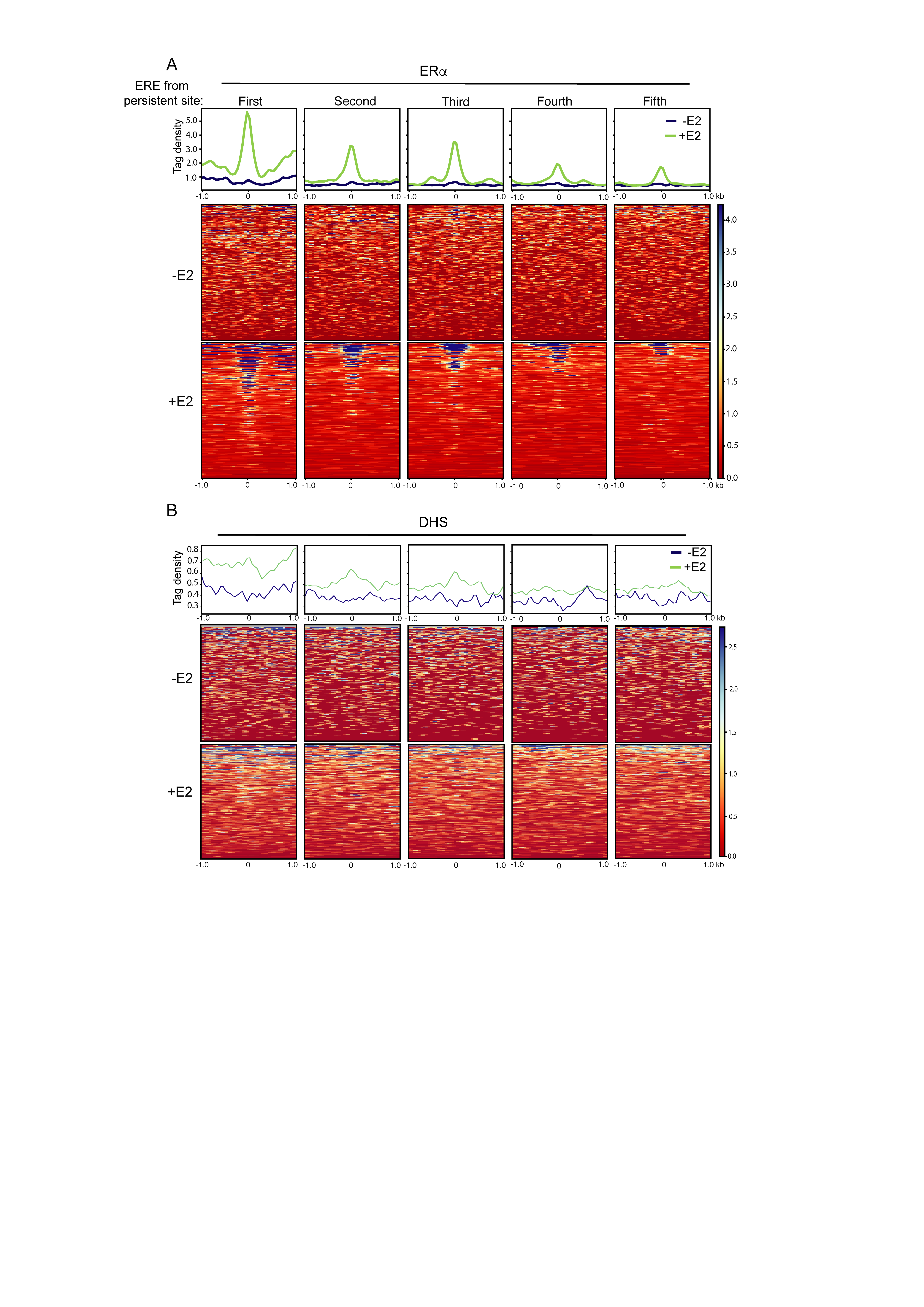

Supplement: S4 Fig — (A) Heatmaps exhibit the loss of ERα binding strength at every 2 consecutive EREs from persistent site (B) Heatmap shows DHS signal on sites in panel A. (TIF) [file pgen.1008516.s004.tif]

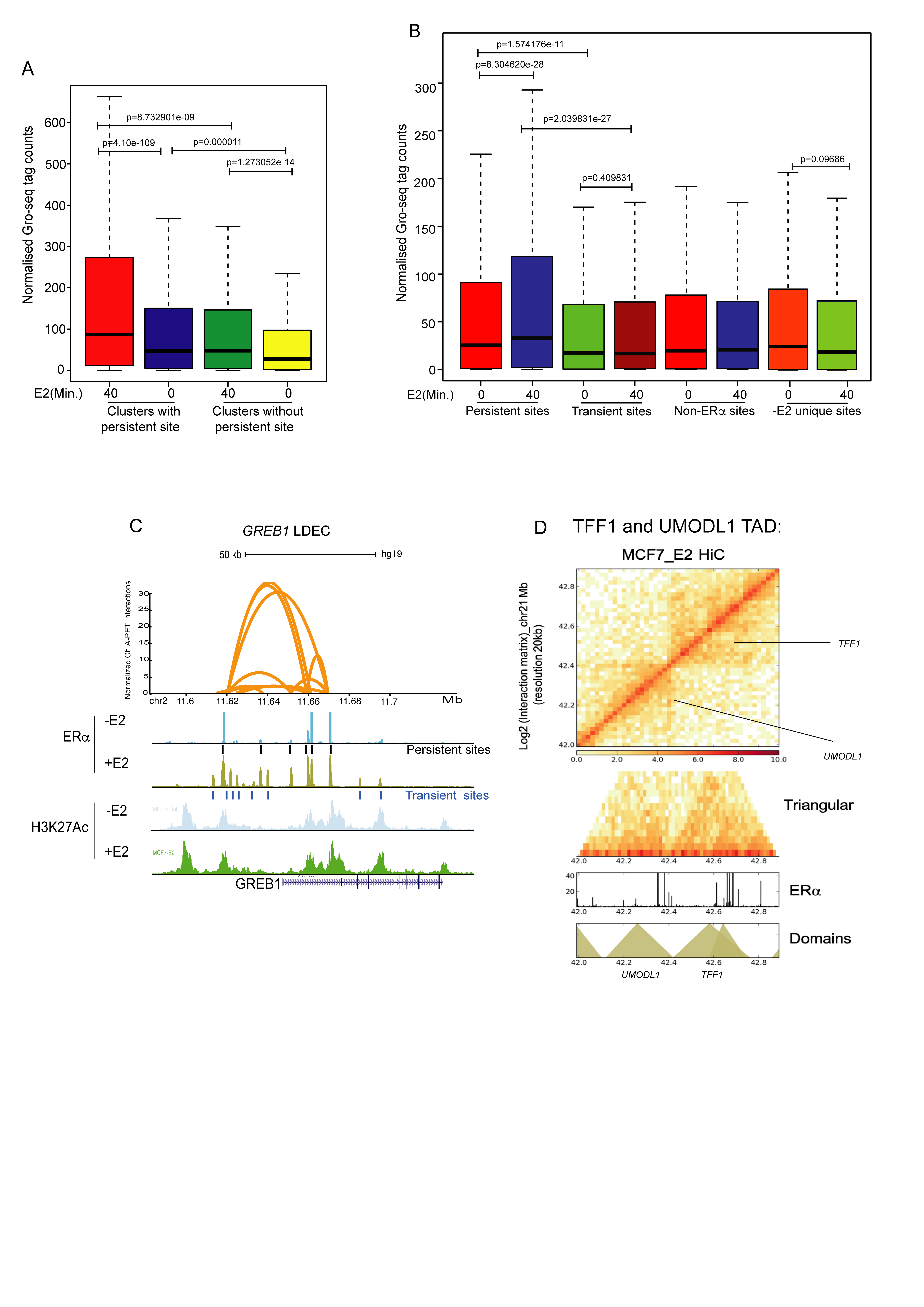

Supplement: S5 Fig — (A) GRO-seq tag count shows the relative higher expression of genes near clusters with a persistent site. (B) GRO-seq tag count shows higher expression of genes closer to persistent vs. transient, random and -E2 unique sites. Note: relative higher expression of these genes (first bar) even in untreated cells. (C) ChIA-PET data plotted from one ERα ChIA-PET replicates on GREB1 LDEC as shown in Fig 3D. (D) TAD structure around TFF1 and UMODL1 genes in MCF-7 cells. (TIF) [file pgen.1008516.s005.tif]

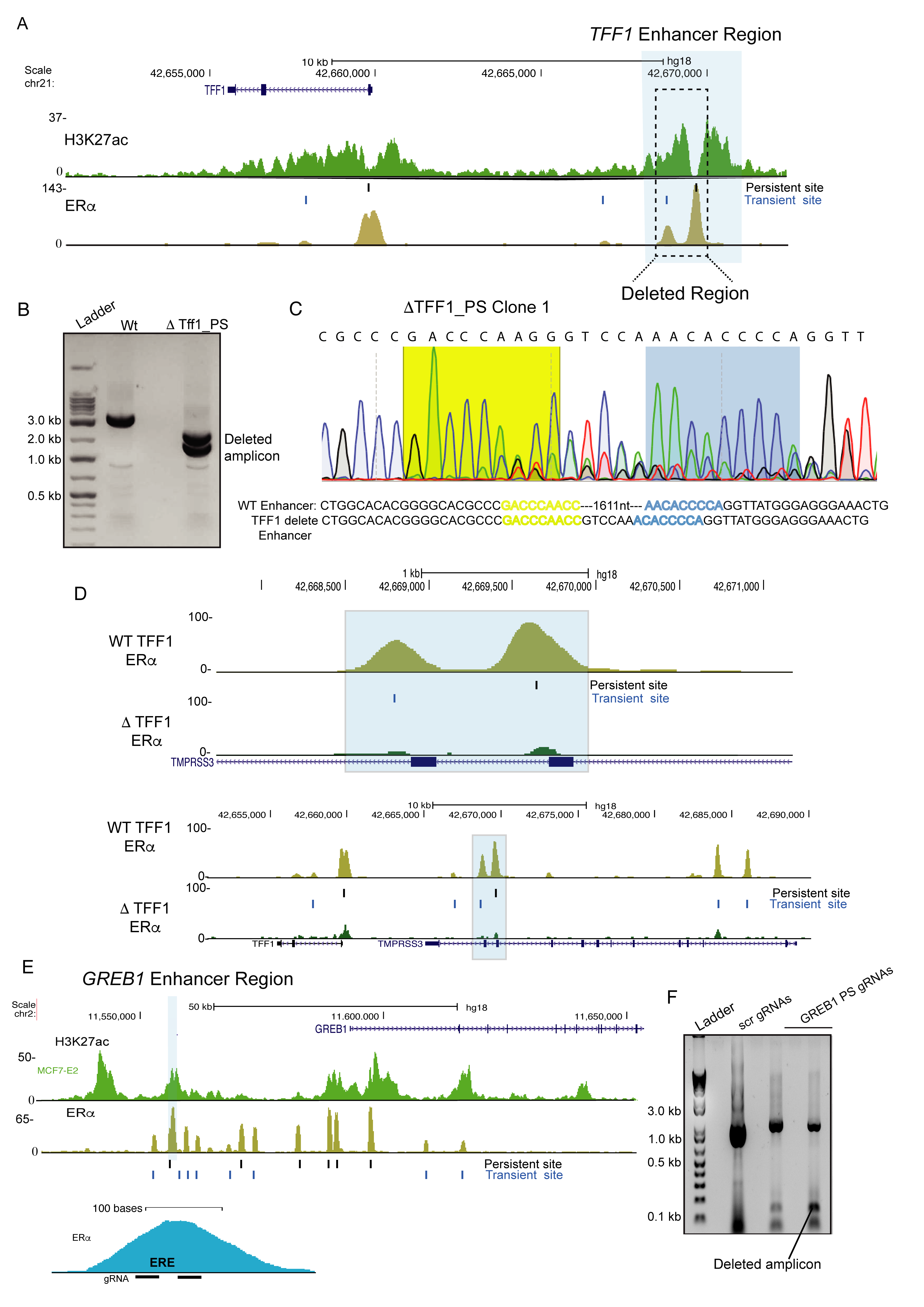

Supplement: S6 Fig — (A) UCSC genome browser snap shot of TFF1 region showing blue highlighted persistent sites. Dashed line box marks the deleted regions. (B) Surveyor assay using the oligos specific for the region outside the deleted PS. Wt genomic DNA exhibits the larger molecular weight amplicon compared to the amplicon from ΔPS-Tff1 genomic DNA. (C) Sanger sequencing chromatogram shows the fusion of yellow and blue highlighted regions in TFF1 delete line, whereas these regions are 1611 nucleotide apart in wild type cells. (D) UCSC genome browser snapshot on TFF1 PS region shows the loss of ERα ChIP-seq peaks in delete cells as compared to wild-type cells (Upper track). Browser snap shot on the wider region around deleted TFF1 site, highlighted region depicts the deleted region (Lower track). (E) UCSC genome browser snap shot of GREB1 region showing blue highlighted persistent site which was blocked by specific gRNAs. (F) gRNA’s cut the specific region within the enhancer as shown by surveyor assay using oligos outside of blocked region, PCR was performed on population of cells after transfection so larger and smaller both amplicons are seen. (TIF) [file pgen.1008516.s006.tif]

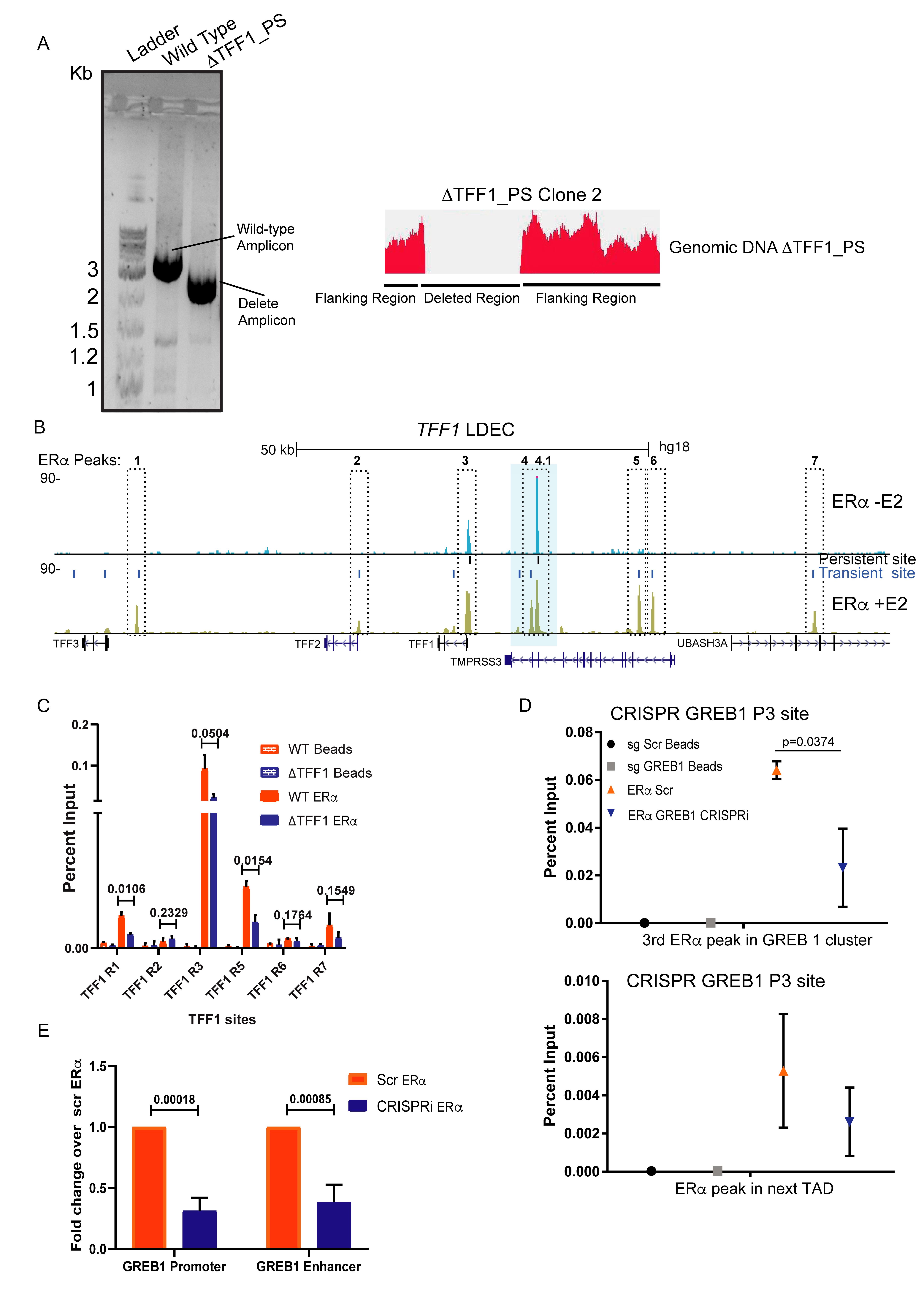

Supplement: S7 Fig — (A) Conformation of TFF1 persistent site deletion in second CRISPR clone (Left panel) and conformation of deletion by sequencing of genomic DNA (Right Panel) (B) UCSC genome bowser track on TFF1 cluster showing ERα ChIP-seq peaks in untreated and treated cells. Highlighted regions depict the deleted region. (C) ChIP-qPCRs shows the loss of ERα binding at different regions of TFF1 cluster upon persistent site deletion in second CRIPSR clone. (D) ChIP-qPCRs confirm the loss of ERα binding at P3 site where gRNAs were targeted in the GREB1 cluster (Upper panel); a modest loss of ERα binding was seen on distal ERα binding site in neighboring TAD (Lower panel). (E) ChIP-qPCRs shows effect in ERα binding strength at GREB1 enhancer region when persistent site near promoter was blocked with gRNAs. (TIF) [file pgen.1008516.s007.tif]

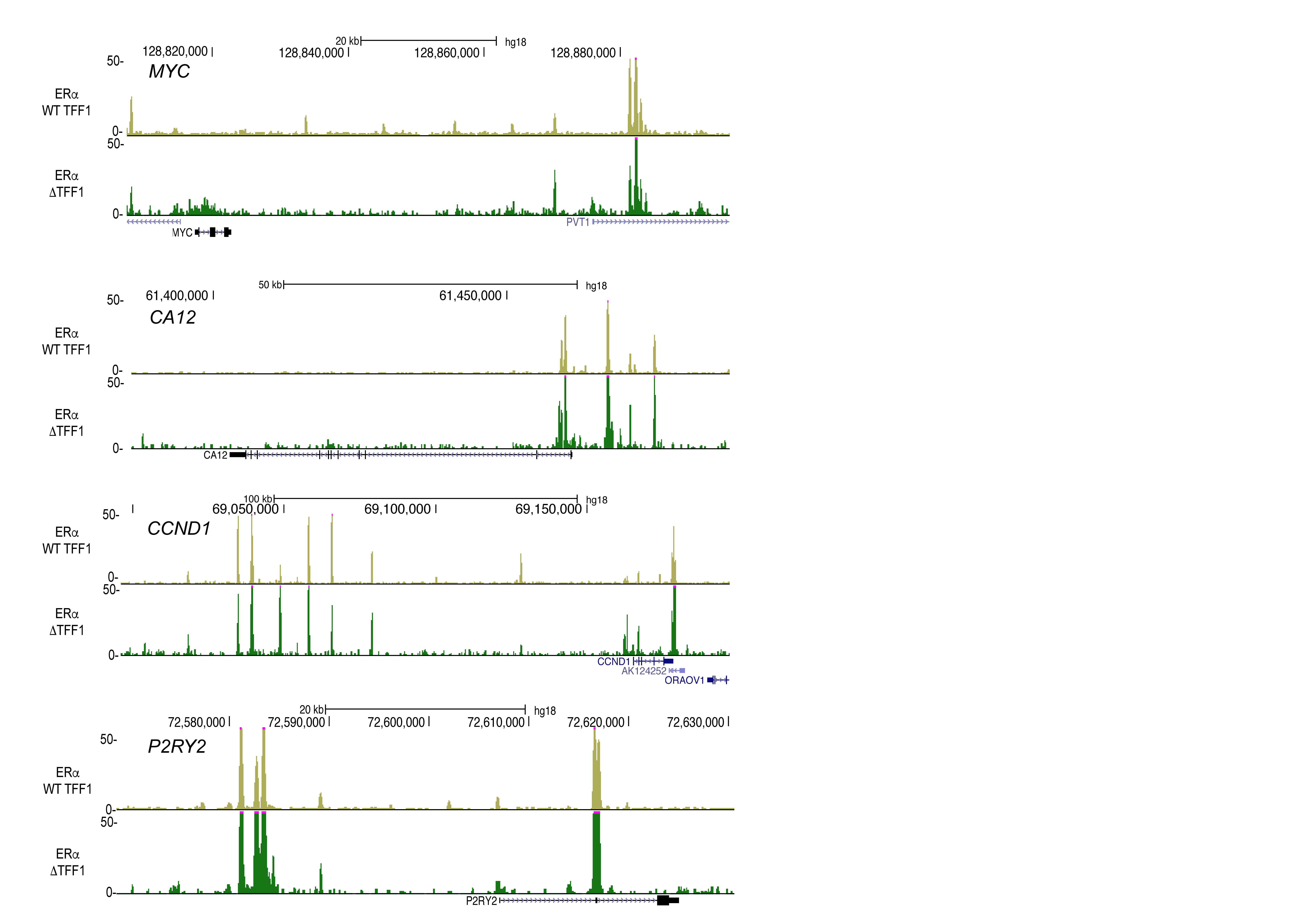

Supplement: S8 Fig — UCSC Genome Browser shots show the occupancy of ERα in WT and ΔTFF1-PS line at various E2 target genes. (TIF) [file pgen.1008516.s008.tif]

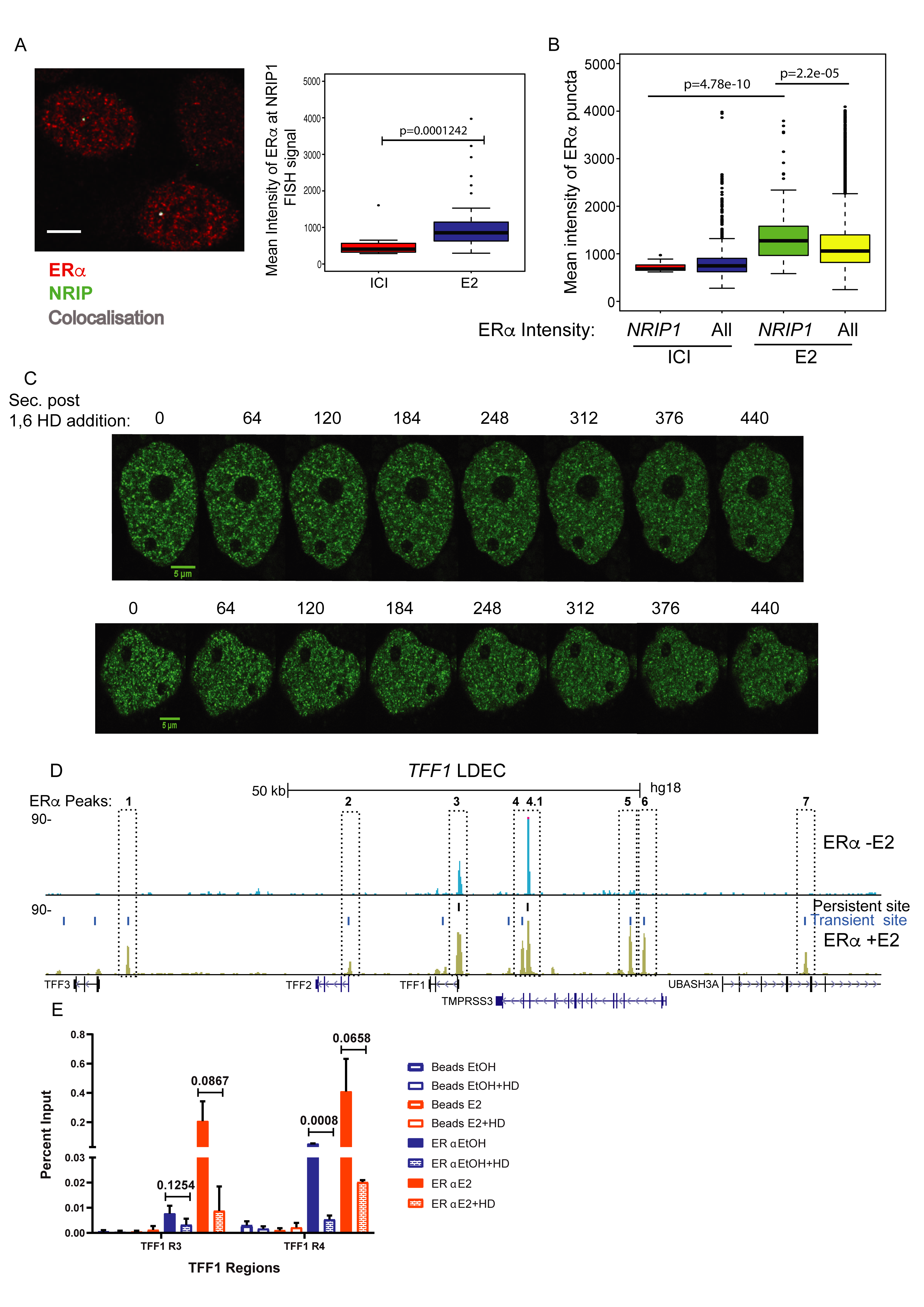

Supplement: S9 Fig — (A) Representative immunoFISH image for NRIP1 (Left panel) and mean ERα intensity on NRIP1 FISH spots (Right panel). (B) Comparison of ERα intensity on all puncta vs. the puncta that overlap with NRIP1 loci by immunoFISH upon ICI and E2 treatment for 1h. (C) Time lapse microscopy images of GFP-ERα upon 1,6-HD treatment in cells treated with E2 for one hour. 0 vs. 440 sec shows significant loss of ERα punctate pattern (D) UCSC genome browser snapshot of TFF1 region showing the ERα ChIP-seq peaks in untreated (Top track) and E2 treated (Bottom track) conditions. Dashed boxes mark the regions on which ERα occupancy was measured upon 1,6-HD treatments. (E) ChIP-qPCRs for ERα on R3 and R4 peaks upon 1,6-HD treatments in E2 treated and untreated conditions. (TIF) [file pgen.1008516.s009.tif]

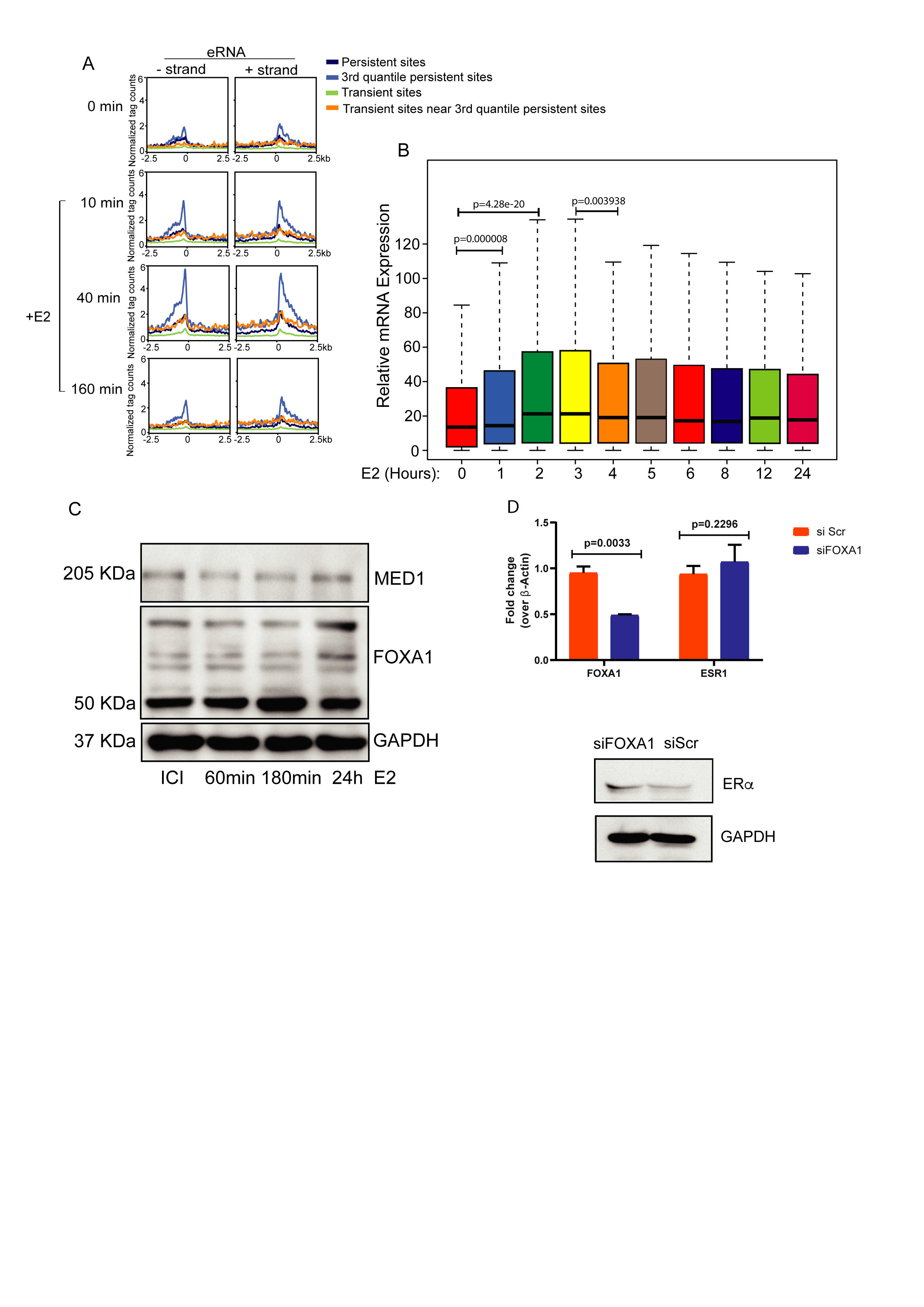

Supplement: S10 Fig — (A) GRO-seq tag counts from plus and minus strands at different categories of ERα peaks during E2 time course. (B) Relative mRNA expression of genes closer to LDEC at different hours of E2 signaling. (C) Immunoblots probed for MED1, FOXA1 and GAPDH in ICI and 60, 180min and 24h post E2 exposure. (D) Levels of ESR1 and FOXA1 by qRT-PCR (upper Panel) and immunoblot for ERα and GAPDH (lower Panel). (JPG) [file pgen.1008516.s010.jpg]
