# Supplementary material for: Ligand dependent gene regulation by transient ERα clustered enhancers
Source: PLoS Genet. 2020 Jan 6;16(1):e1008516. doi: 10.1371/journal.pgen.1008516 (PMC6975561; doi:10.1371/journal.pgen.1008516)
Supplement: S1 Table — (PDF) [file pgen.1008516.s013.pdf]

TableS1: 5C oligos used in the study:

Chr21 Forward Oligo ID	Sequences (5'-3')	Modification
5C_chr21-BamHI_FOR_2	TAATACGACTCACTATAGCCGGGCGGTGTGGCTGGGGGCTGGAGGCTGGA	
5C_chr21-BamHI_FOR_4	TAATACGACTCACTATAGCCACTTATTTTAAAAAGAACCCATAGAATGGA	
5C_chr21-BamHI_FOR_101	TAATACGACTCACTATAGCCGTGCTCTCCAGCTACACAGACTTCTCTGGA	
5C_chr21-BamHI_FOR_103	TAATACGACTCACTATAGCCACAGGGGTTAGCTTGGATTTTACTGCGGGA	
5C_chr21-BamHI_FOR_106	TAATACGACTCACTATAGCCTCCGTGGGTCGCGAAAGGCGCCGCCCAGGA	
5C_chr21-BamHI_FOR_108	TAATACGACTCACTATAGCCTATGCTGCCACTTCTCTAAGATAATAGGGA	
5C_chr21-BamHI_FOR_120	TAATACGACTCACTATAGCCTTGTTCTAAGAAGGTAGTAATTAGAGTGGA	
5C_chr21-BamHI_FOR_123	TAATACGACTCACTATAGCCATCGACTGAAACCGGTTACCTGGGTTGGGA	
5C_chr21-BamHI_FOR_125	TAATACGACTCACTATAGCCGACGGCACCAGAATCCCAGATCCTCAAGGA	
5C_chr21-BamHI_FOR_127	TAATACGACTCACTATAGCCAAGCTCTCCTGACTTGCAATGTCTCCAGGA	
5C_chr21-BamHI_FOR_129	TAATACGACTCACTATAGCCACGGAAGAAACTAGGGTAACACTGGCAGGA	
5C_chr21-BamHI_FOR_170	TAATACGACTCACTATAGCCATCTGCCTTGGTTTAATGTAGACGAAGGGA	
5C_chr21-BamHI_FOR_173	TAATACGACTCACTATAGCCGCAAGCCACATGTAGGAGAATGATGATGGA	
5C_chr21-BamHI_FOR_191	TAATACGACTCACTATAGCCCATCCTACTTCAGAGGCTGAGGCAGGAGGA	
5C_chr21-BamHI_FOR_193	TAATACGACTCACTATAGCCTTCCTGATGTTCCAGGAGAGTACGGTTGGA	
5C_chr21-BamHI_FOR_299	TAATACGACTCACTATAGCCGTGGACACGCATCACAGTCTTGCCAGCGGA	
5C_chr21-BamHI_FOR_301	TAATACGACTCACTATAGCCCCTCCTTACTCTGGACTTTTCCAGGTAGGA	
5C_chr21-BamHI_FOR_341	TAATACGACTCACTATAGCCAGCTTTTGTGTTTTGCTCTAATCAGCAGGA	
5C_chr21-BamHI_FOR_481	TAATACGACTCACTATAGCCCAAGTCCTCGACAGCGGACAGCCTGCCGGA	
5C_chr21-BamHI_FOR_483	TAATACGACTCACTATAGCCTTAATTTTTGTATAAGGTTTAAGGAAGGGA	
5C_chr21-BamHI_FOR_660	TAATACGACTCACTATAGCCTTGTGGCTATTAACGAATTGAGGGGCAGGA	
5C_chr21-BamHI_FOR_662	TAATACGACTCACTATAGCCTGCTGGTGGTTTCCTGTGAATAAATTTGGA	
5C_chr21-BamHI_FOR_663	TAATACGACTCACTATAGCCACGCTACTTAGGAGGCTGAGGTAAGGAGGA	
5C_chr21-BamHI_FOR_676	TAATACGACTCACTATAGCCCTCCCTTTTCCTCCCTTGCTGTCTCTTGGA	

5C_chr21-BamHI_FOR_818	TAATACGACTCACTATAGCCTAACGCCGTTCTTGTCAATATCACCAAGGA	
5C_chr21-BamHI_FOR_820	TAATACGACTCACTATAGCCTTGGCAAGTATGTATCTAGTCACACAAGGA	
5C_chr21-BamHI_FOR_1377	TAATACGACTCACTATAGCCGAAAGCCTCCTGCAAACTCAGTCCCATGGA	
5C_chr21-BamHI_FOR_1379	TAATACGACTCACTATAGCCCCTGGAAAGTAGGCAATCAGGAATATAGGA	
5C_chr21-BamHI_FOR_1382	TAATACGACTCACTATAGCCAATCGATGAAGGGAGGCTGAGGCAGGAGGA	
5C_chr21-BamHI_FOR_1384	TAATACGACTCACTATAGCCTGTTTTAAAATCTGAGTTAGTGCGTCTGGA	
5C_chr21-BamHI_FOR_1386	TAATACGACTCACTATAGCCAATCTCTTCCTTTTCTTCTGAGATCCAGGA	
5C_chr21-BamHI_FOR_1389	TAATACGACTCACTATAGCCGATTTCCTTCTGCCTTTGGGTCCTTTTGGA	
5C_chr21-BamHI_FOR_1391	TAATACGACTCACTATAGCCCTGAACTCCCTTTGTTTACTCCCTATCGGA	
5C_chr21-BamHI_FOR_1393	TAATACGACTCACTATAGCCTAGCTTTGAATAGTATTTATTACTTATGGA	
5C_chr21-BamHI_FOR_1395	TAATACGACTCACTATAGCCGTTAACATTTTATCACTACTGTCAGGAGGA	
5C_chr21-BamHI_FOR_1402	TAATACGACTCACTATAGCCCTCAAGTTTATGTTCAGTATGAAAGAAGGA	
5C_chr21-BamHI_FOR_1404	TAATACGACTCACTATAGCCCATGTTTGAAGTGTGAGTTGCCCAAGTGGA	
5C_chr21-BamHI_FOR_1610	TAATACGACTCACTATAGCCACTCAATGGCACCAAGGCATTCATGAGGGA	
5C_chr21-BamHI_FOR_1612	TAATACGACTCACTATAGCCACGTCTCCCTCAAGAGACTCTGGGGAAGGA	
5C_chr21-BamHI_FOR_1615	TAATACGACTCACTATAGCCGCCGGAATCTTATCTATCTTCCCTGCTGGA	
5C_chr21-BamHI_FOR_1617	TAATACGACTCACTATAGCCATTGGAAGCCAGAAGTATAAGAAAAATGGA	
5C_chr21-BamHI_FOR_2069	TAATACGACTCACTATAGCCTCCTCCTGATGGAAGGGGCAAGGTTCTGGA	
5C_chr21-BamHI_FOR_2072	TAATACGACTCACTATAGCCCTCGATGAGCCCATCTACTGGTCCTTTGGA	
5C_chr21-BamHI_FOR_2075	TAATACGACTCACTATAGCCCCTACCCTAGGGTGTGGCCCTCTTCTGGGA	
5C_chr21-BamHI_FOR_2238	TAATACGACTCACTATAGCCAACTCTAATGTATTGAAGCCTATTAATGGA	
5C_chr21-BamHI_FOR_2240	TAATACGACTCACTATAGCCGGCTCCTTTTCCCATAGATGAACCGGTGGA	
5C_chr21-BamHI_FOR_2243	TAATACGACTCACTATAGCCCGGAACACATTGCGAGATATGCAGACAGGA	
5C_chr21-BamHI_FOR_2245	TAATACGACTCACTATAGCCGAGGCAGATGAGAAGCTAGGAGTCACAGGA	
5C_chr21-BamHI_FOR_2272	TAATACGACTCACTATAGCCTACTTTTCTGCTGTGAGAATGTAAGATGGA	
5C_chr21-BamHI_FOR_2274	TAATACGACTCACTATAGCCTATCCGCACTGCCACCGTGCCTGGCCGGGA	
5C_chr21-BamHI_FOR_2275	TAATACGACTCACTATAGCCGCTTGCCACCATTGTTTTTCAAACCTAGGA	

5C_chr21-BamHI_FOR_2278	TAATACGACTCACTATAGCCGGTTGTTAAAGGGAGGCCGAGGCGGGTGGA	
5C_chr21-BamHI_FOR_2280	TAATACGACTCACTATAGCCCCCCTGCCCGTCCATCTTTTTGCCTTGGGA	
5C_chr21-BamHI_FOR_2284	TAATACGACTCACTATAGCCCTTTGGGTTTGATTCCTCCCATTGCTGGGA	
5C_chr21-BamHI_FOR_2286	TAATACGACTCACTATAGCCTATAGTCCCAGCTACTTGGGAGGCTAAGGA	
5C_chr21-BamHI_FOR_2287	TAATACGACTCACTATAGCCTTAATTTTTATATAAGGTGTAAGGAAGGGA	
5C_chr21-BamHI_FOR_2289	TAATACGACTCACTATAGCCAGTGTTACAGTTTCTCCACATCATCCAGGA	
5C_chr21-BamHI_FOR_2301	TAATACGACTCACTATAGCCGATCCTGTGCAGGTCAGCCTCGGAAAAGGA	
5C_chr21-BamHI_FOR_2304	TAATACGACTCACTATAGCCCCCTGCTTTCAAACTCCTGACCTCAAGGGA	
5C_chr21-BamHI_FOR_2307	TAATACGACTCACTATAGCCATATTCAGCCATTAACATGGATGAATTGGA	
5C_chr21-BamHI_FOR_2309	TAATACGACTCACTATAGCCACGAGGGAGAGAGGCCTCCTCCAGGGAGGA	
5C_chr21-BamHI_FOR_2314	TAATACGACTCACTATAGCCATTTTACATCCCTGTTACATTTCTCTAGGA	
5C_chr21-BamHI_FOR_2316	TAATACGACTCACTATAGCCGAAGTCGTTGAGAGCCACCCAAGAGGTGGA	
5C_chr21-BamHI_FOR_2317	TAATACGACTCACTATAGCCAGGCCACGACGGCGCCAGAGGAAACAGGGA	
5C_chr21-BamHI_FOR_2322	TAATACGACTCACTATAGCCTGGAAAAAGTTATGAGTAAGCTACCACGGA	
5C_chr21-BamHI_FOR_2422	TAATACGACTCACTATAGCCCTCCGCTCCTCAAGGACCAGACAGCAAGGA	
5C_chr21-BamHI_FOR_2424	TAATACGACTCACTATAGCCGGTCGTCTTTGGGAGGTCAAGGCAGGAGGA	
5C_chr21-BamHI_FOR_2443	TAATACGACTCACTATAGCCAGATCTTATTCATGAGTCTATATTTTGGGA	
5C_chr21-BamHI_FOR_2447	TAATACGACTCACTATAGCCCCAGCCAGCAGAAGACCAGTCTGCTTCGGA	
5C_chr21-BamHI_FOR_2449	TAATACGACTCACTATAGCCTGGGCCTAATAAGGGAGTCATATTAGAGGA	
5C_chr21-BamHI_FOR_2460	TAATACGACTCACTATAGCCCTCGAACCTCAGTGACATGCACCGAGTGGA	
5C_chr21-BamHI_FOR_2462	TAATACGACTCACTATAGCCTATAAATTTAATAACACACACATAAAAGGA	
5C_chr21-BamHI_FOR_2494	TAATACGACTCACTATAGCCGAAGAAGGTGTTGGGGCATCCACTTGGGGA	
5C_chr21-BamHI_FOR_2496	TAATACGACTCACTATAGCCTTGGCATTTGCACTGGATTATCGGGTAGGA	
5C_chr21-BamHI_FOR_2521	TAATACGACTCACTATAGCCGGCATAAGAGCACGTGTTGAATGAGATGGA	
5C_chr21-BamHI_FOR_2523	TAATACGACTCACTATAGCCATCCACAACAAGCCCTTACTGGAAGGGGGA	
5C_chr21-BamHI_FOR_2614	TAATACGACTCACTATAGCCAACTAATCTGTATGATATTATAATGGTGGA	
5C_chr21-BamHI_FOR_2616	TAATACGACTCACTATAGCCGCAATTCTGAATGATTAGATTGAAATTGGA	

5C_chr21-BamHI_FOR_2618	TAATACGACTCACTATAGCCGGAAGCACGTGGGCAGCATCTAATGAGGGA	
5C_chr21-BamHI_FOR_2620	TAATACGACTCACTATAGCCTAACAGATCCAAAATGGACTTCCAGGAGGA	
5C_chr21-BamHI_FOR_2666	TAATACGACTCACTATAGCCTACTTCATAAAGGGCAGCCCTAGTGCTGGA	
5C_chr21-BamHI_FOR_2668	TAATACGACTCACTATAGCCTCGAAAGAGAGACCCCATCGCTGCCTGGGA	
5C_chr21-BamHI_FOR_2671	TAATACGACTCACTATAGCCAGGATATCCAGCAGGGTGTCTGGGCTGGGA	
5C_chr21-BamHI_FOR_2673	TAATACGACTCACTATAGCCTGTCTCGATGATGTCATCTGAGACTCTGGA	
5C_chr21-BamHI_FOR_2736	TAATACGACTCACTATAGCCTCTCATTTTGGCGAGTGGAAAGCGATGGGA	
5C_chr21-BamHI_FOR_2738	TAATACGACTCACTATAGCCAGTGAGGGCTCTTCCCTCCTCTGGCCTGGA	
5C_chr21-BamHI_FOR_2741	TAATACGACTCACTATAGCCAGGCTGGTGTTAGGAAATGTCTACAAGGGA	
5C_chr21-BamHI_FOR_2743	TAATACGACTCACTATAGCCCACCACTCTATCAGGACAGCCAGCAGCGGA	
5C_chr21-BamHI_FOR_2745	TAATACGACTCACTATAGCCGACCAACCCCACTGAAGGTTCTAGGGAGGA	
5C_chr21-BamHI_FOR_2779	TAATACGACTCACTATAGCCATCCTAGATTCCTTTTAATCAGTCTATGGA	
5C_chr21-BamHI_FOR_2782	TAATACGACTCACTATAGCCGACATTGCTCGTACTCCTGGGCTCAAGGGA	
5C_chr21-BamHI_FOR_2821	TAATACGACTCACTATAGCCGGTCTACACAGGGATGCCCTGTGGACAGGA	
5C_chr21-BamHI_FOR_2823	TAATACGACTCACTATAGCCACTCACTGAGGAAGGAACAAGGATGCGGGA	
5C_chr21-BamHI_FOR_2872	TAATACGACTCACTATAGCCTTCAAAATGTAGCTCACAGACCTGTAGGGA	
5C_chr21-BamHI_FOR_2873	TAATACGACTCACTATAGCCTGCGAATCCCAAATTAATTTTATATCAGGA	
5C_chr21-BamHI_FOR_2874	TAATACGACTCACTATAGCCTACGCAGTTGCCTCTCTGAAGACAAAGGGA	
5C_chr21-BamHI_FOR_2877	TAATACGACTCACTATAGCCCTTCCGAAGGCATACCAGTTCAGACTTGGA	
5C_chr21-BamHI_FOR_3065	TAATACGACTCACTATAGCCTCCGCACGCAGTGCATCCCATATTGGAGGA	
5C_chr21-BamHI_FOR_3069	TAATACGACTCACTATAGCCAACTGGTTGTGTTTGTCTAAAGACCTGGGA	
5C_chr21-BamHI_FOR_3078	TAATACGACTCACTATAGCCTACAGCCACTAGGAACCCACAAGGACAGGA	
5C_chr21-BamHI_FOR_3079	TAATACGACTCACTATAGCCTTCTGAGACCCTCAGGCTCATAGAACTGGA	
5C_chr21-BamHI_FOR_3082	TAATACGACTCACTATAGCCGGCTTAACCTAAGGAGGAGATCCTGGGGGA	
5C_chr21-BamHI_FOR_3099	TAATACGACTCACTATAGCCAGACACTAGGCCAGACTGCAGGCGGCAGGA	
5C_chr21-BamHI_FOR_3101	TAATACGACTCACTATAGCCGTTCCCACGGTTGACAGGGAAGCCCTGGGA	
5C_chr21-BamHI_FOR_3161	TAATACGACTCACTATAGCCATGTGTTTTCTGCGTAACAACGAGATGGGA	

5C_chr21-BamHI_FOR_3163	TAATACGACTCACTATAGCCCGGTGGTCGACCTGGGTGCCACGAAGGGGA	
5C_chr21-BamHI_FOR_3165	TAATACGACTCACTATAGCCTCCGGGCGCTGTCGTGGTGGAGAACGCGGA	
5C_chr21-BamHI_FOR_3167	TAATACGACTCACTATAGCCCGTCCTTTGTGCCTGTGGGAACTACGGGGA	
5C_chr21-BamHI_FOR_3169	TAATACGACTCACTATAGCCTCTGACTTTCTAGTAACCACCGATTCCGGA	
5C_chr21-BamHI_FOR_3171	TAATACGACTCACTATAGCCCGTCTCTGAGAGAAGGGCCGTGAGTGAGGA	
5C_chr21-BamHI_FOR_3174	TAATACGACTCACTATAGCCCTTCGGCGGGCTTGGGAGGTTTGTACAGGA	
5C_chr21-BamHI_FOR_3176	TAATACGACTCACTATAGCCGATCGAGGCTTATGTTCAGCACCCCAGGGA	
5C_chr21-BamHI_FOR_3203	TAATACGACTCACTATAGCCGTACTCACCAGGCGGGTGGGCCTGCAGGGA	
5C_chr21-BamHI_FOR_3206	TAATACGACTCACTATAGCCCGTGGACGGAGGCCCTGCATCCAGGGAGGA	
5C_chr21-BamHI_FOR_3229	TAATACGACTCACTATAGCCCTCTCGTGGCTTTGACTCCCAGAGCATGGA	
5C_chr21-BamHI_FOR_3231	TAATACGACTCACTATAGCCCTCCCAGGGAGGAGTTTGGGAGGAAGTGGA	
5C_chr21-BamHI_FOR_3234	TAATACGACTCACTATAGCCCGCGGCGTCCCACCTCCATCTGCTCGTGGA	
5C_chr21-BamHI_FOR_3235	TAATACGACTCACTATAGCCCCCTGGTCTCAAACTAATGGCCTCAAGGGA	
5C_chr21-BamHI_FOR_3237	TAATACGACTCACTATAGCCTGTGCTTTTCTCATAGTGAAGGGAAAGGGA	
5C_chr21-BamHI_FOR_3238	TAATACGACTCACTATAGCCCGGAGCCCATCACAGAGCAGTGACTCTGGA	
5C_chr21-BamHI_FOR_3240	TAATACGACTCACTATAGCCATGTCTTGTTCACAGACAAGGAGACTGGGA	
5C_chr21-BamHI_FOR_3350	TAATACGACTCACTATAGCCTCTTTACTGCATCCTGTTTTATCAGCAGGA	
5C_chr21-BamHI_FOR_3352	TAATACGACTCACTATAGCCCTACCAACCATGGTTTGTGCTCTGGCAGGA	
5C_chr21-BamHI_FOR_3353	TAATACGACTCACTATAGCCCTCTAACAGTGCATGCTCTTTGGGTGAGGA	
5C_chr21-BamHI_FOR_3355	TAATACGACTCACTATAGCCGTCAGACATTGTCGCCTGGGACAATTAGGA	
5C_chr21-BamHI_FOR_3398	TAATACGACTCACTATAGCCCTGGGGTACGAGCCAGGCCAGGAGGCAGGA	
5C_chr21-BamHI_FOR_3402	TAATACGACTCACTATAGCCGGGAACTCCGACGTGGCCTGGGGTGGGGGA	
5C_chr21-BamHI_FOR_3404	TAATACGACTCACTATAGCCCCTCTAAGGTGGCGCTTGCTTTTTAAGGGA	
5C_chr21-BamHI_FOR_3406	TAATACGACTCACTATAGCCGGTCGTGAAAGGGAGGCCGAGGTGGGTGGA	
5C_chr21-BamHI_FOR_3454	TAATACGACTCACTATAGCCGAATGGGAGAAAATATTCGCAAACTATGGA	
5C_chr21-BamHI_FOR_3456	TAATACGACTCACTATAGCCGATGGAAGGTCCCTGGAAGACTAAGCTGGA	
5C_chr21-BamHI_FOR_3466	TAATACGACTCACTATAGCCGTGGTGGCTTAATGGCTGCATTAGATAGGA	

5C_chr21-BamHI_FOR_3468	TAATACGACTCACTATAGCCAGAGGAGCATCAGCCTCCCAAGTAGCTGGA	
5C_chr21-BamHI_FOR_3470	TAATACGACTCACTATAGCCTGACCGTAAGCCTGGAAAGGGAGGGGCGGA	
5C_chr21-BamHI_FOR_3472	TAATACGACTCACTATAGCCCAGAAAGGTTAGAGGCCAGGCAGTGCTGGA	
5C_chr21-BamHI_FOR_3553	TAATACGACTCACTATAGCCCTAGAGGCAGAGCCAGGATCTCCCCCAGGA	
5C_chr21-BamHI_FOR_3555	TAATACGACTCACTATAGCCCCCCTCGGGACCTTGGGAGCGGGCCCAGGA	
5C_chr21-BamHI_FOR_3569	TAATACGACTCACTATAGCCCCTTTGCCACATGAGGTAACATTCACAGGA	
5C_chr21-BamHI_FOR_3571	TAATACGACTCACTATAGCCCGAGGTGTCGCGGACAGCATTCTGCAAGGA	
5C_chr21-BamHI_FOR_3724	TAATACGACTCACTATAGCCGGTAGTGCCGCCAATATGTGCAAGCCAGGA	
5C_chr21-BamHI_FOR_3726	TAATACGACTCACTATAGCCCCGACGTCTCGAGCTCCTGGACTCAAGGGA	
5C_chr21-BamHI_FOR_3784	TAATACGACTCACTATAGCCACCACAGTCCCACATGGTGTTGCCCCAGGA	
5C_chr21-BamHI_FOR_3786	TAATACGACTCACTATAGCCCGACGACCCTGCTGGGAGAGGCGGGAGGGA	
5C_chr21-BamHI_FOR_3788	TAATACGACTCACTATAGCCTCCCCTCGTACTCCCCTGCTGTGCCCAGGA	
5C_chr21-BamHI_FOR_3795	TAATACGACTCACTATAGCCGCGCCCCTGGAGGACGTGGTGGCGGCCGGA	
5C_chr21-BamHI_FOR_3802	TAATACGACTCACTATAGCCAGGAGATTTGACAGGAGAAGCATGATGGGA	
5C_chr21-BamHI_FOR_3804	TAATACGACTCACTATAGCCTCCGAATGGGCGCTGCAGCTCGCTCTCGGA	
5C_chr21-BamHI_FOR_3808	TAATACGACTCACTATAGCCGTCGAGACGCGCCCAAACGGCCAGGCTGGA	
5C_chr21-BamHI_FOR_3810	TAATACGACTCACTATAGCCCTTTAGATCTTCCCAAAGGTGGTGCTGGGA	
5C_chr21-BamHI_FOR_3914	TAATACGACTCACTATAGCCCGGTCTCGTGGCCAGAGGTGAGGAAGGGGA	
5C_chr21-BamHI_FOR_3916	TAATACGACTCACTATAGCCGAAAAAAGAGCAAGAGAGAAAGCCCAAGGA	
5C_chr21-BamHI_FOR_3921	TAATACGACTCACTATAGCCCGTACGCACCACACCTGGCACCTCTTTGGA	
5C_chr21-BamHI_FOR_3924	TAATACGACTCACTATAGCCCCGAAATGAGCAACACACACCAACCTGGGA	
5C_chr21-BamHI_FOR_3997	TAATACGACTCACTATAGCCGTGTCGGATGCCCTGAATCGCCCTTGTGGA	
5C_chr21-BamHI_FOR_3999	TAATACGACTCACTATAGCCGACGTCGTAGGACCTCCTGGGCTCAAGGGA	
**Chr21 Reverse Oligos ID**	**Sequences (5'-3')**	
5C_chr21-BamHI_REV_3	TCCTTAAGGCCCCCAACCCGGAGGGGTGCTTCCCTTTAGTGAGGGTTAATA	5'Phosphor
5C_chr21-BamHI_REV_5	TCCAACTAGGTCTGTCCGCTTCCGAGGCGCTCCCTTTAGTGAGGGTTAATA	5'Phosphor

5C_chr21-BamHI_REV_102	TCCAACTAGGAGTTGGATAAGTAAATTATATCCCTTTAGTGAGGGTTAATA	5'Phosphor
5C_chr21-BamHI_REV_105	TCCTCTTAGTATTAAGATTTCTTAAAAAGCTCCCTTTAGTGAGGGTTAATA	5'Phosphor
5C_chr21-BamHI_REV_107	TCCGCGCGGCTTCCTCCTGAGGCCCACTGTTCCCTTTAGTGAGGGTTAATA	5'Phosphor
5C_chr21-BamHI_REV_118	TCCCTAGTGTATACTTCCCAAACACTATTATCCCTTTAGTGAGGGTTAATA	5'Phosphor
5C_chr21-BamHI_REV_121	TCCCTCACTATGTTGCCCAGGATGATCTTCTCCCTTTAGTGAGGGTTAATA	5'Phosphor
5C_chr21-BamHI_REV_124	TCCCTGATGGGTCTATGCATGTATGCAAAATCCCTTTAGTGAGGGTTAATA	5'Phosphor
5C_chr21-BamHI_REV_126	TCCACTAGGTGAAGCCAGCTGGACAGGACTTCCCTTTAGTGAGGGTTAATA	5'Phosphor
5C_chr21-BamHI_REV_128	TCCACACCCACATGACGCAGCGAGAGGTAGTCCCTTTAGTGAGGGTTAATA	5'Phosphor
5C_chr21-BamHI_REV_172	TCCAATCAGCATAATGTCATCAATGTAATGTCCCTTTAGTGAGGGTTAATA	5'Phosphor
5C_chr21-BamHI_REV_174	TCCTCCCACCTCAGCCTCCTGTCATCGTCCTCCCTTTAGTGAGGGTTAATA	5'Phosphor
5C_chr21-BamHI_REV_190	TCCACCCGCGTGACCCAAACTGAAGGGTGATCCCTTTAGTGAGGGTTAATA	5'Phosphor
5C_chr21-BamHI_REV_192	TCCCTAGCTTACCCAAAGCCTCCAAAGGTTTCCCTTTAGTGAGGGTTAATA	5'Phosphor
5C_chr21-BamHI_REV_297	TCCCAGAGGTCCATGATGGGAGTGTCCACGTCCCTTTAGTGAGGGTTAATA	5'Phosphor
5C_chr21-BamHI_REV_300	TCCATTGCTAGGGACCTAGTGCAATCCTTTTCCCTTTAGTGAGGGTTAATA	5'Phosphor
5C_chr21-BamHI_REV_340	TCCACTTCCCAGATCGTTCATGTGATTTTGTCCCTTTAGTGAGGGTTAATA	5'Phosphor
5C_chr21-BamHI_REV_342	TCCCACTTATGTAGAAATTTATATAGTTGGTCCCTTTAGTGAGGGTTAATA	5'Phosphor
5C_chr21-BamHI_REV_482	TCCGGCAGGGTGTCCACTGTGGAAGACTAGTCCCTTTAGTGAGGGTTAATA	5'Phosphor
5C_chr21-BamHI_REV_484	TCCAATAAGGTCAGTTCATTTAAAATTCAATCCCTTTAGTGAGGGTTAATA	5'Phosphor
5C_chr21-BamHI_REV_659	TCCAGGCAAGTCCCACTGTACTTTTTAGGATCCCTTTAGTGAGGGTTAATA	5'Phosphor
5C_chr21-BamHI_REV_661	TCCTTAGTGGCACAAGTGAACATAGAAAACTCCCTTTAGTGAGGGTTAATA	5'Phosphor
5C_chr21-BamHI_REV_664	TCCATGTAATCCTTATCCATGTAAGACAGGTCCCTTTAGTGAGGGTTAATA	5'Phosphor
5C_chr21-BamHI_REV_677	TCCATAGTTAGATTAGTTAGATTTAAGTGCTCCCTTTAGTGAGGGTTAATA	5'Phosphor
5C_chr21-BamHI_REV_819	TCCATACAACTTCCTCATGAAGTCCTACTTTCCCTTTAGTGAGGGTTAATA	5'Phosphor
5C_chr21-BamHI_REV_1378	TCCTCCCTCAGGACCCAAACACCAGGGTGGTCCCTTTAGTGAGGGTTAATA	5'Phosphor
5C_chr21-BamHI_REV_1380	TCCCTTATGTTGTAATTAGTGACCTTATAATCCCTTTAGTGAGGGTTAATA	5'Phosphor
5C_chr21-BamHI_REV_1381	TCCTTCAGGACCTCCGTGGCCAAGAGTGTTTCCCTTTAGTGAGGGTTAATA	5'Phosphor
5C_chr21-BamHI_REV_1383	TCCCTCGAAGCCAGGAATTCATGACCTCGGTCCCTTTAGTGAGGGTTAATA	5'Phosphor

5C_chr21-BamHI_REV_1385	TCCAGAAGGCTATCACACTGGCCCAGACGGTCCCTTTAGTGAGGGTTAATA	5'Phosphor
5C_chr21-BamHI_REV_1387	TCCTTCGTGTGGCTAAAACCAGTTCTAACATCCCTTTAGTGAGGGTTAATA	5'Phosphor
5C_chr21-BamHI_REV_1390	TCCCCTCAATATTTGCATCTACTTAAGAAATCCCTTTAGTGAGGGTTAATA	5'Phosphor
5C_chr21-BamHI_REV_1392	TCCATGTCTTAATCACCAGACCTTGCGAATTCCCTTTAGTGAGGGTTAATA	5'Phosphor
5C_chr21-BamHI_REV_1394	TCCTTCACACAGAGTCTCCACTGGGCGTGATCCCTTTAGTGAGGGTTAATA	5'Phosphor
5C_chr21-BamHI_REV_1403	TCCTATTTATCACATCCAGGAATTAGTCATTCCCTTTAGTGAGGGTTAATA	5'Phosphor
5C_chr21-BamHI_REV_1406	TCCAATAAGTACATGCCCAACCATATGATGTCCCTTTAGTGAGGGTTAATA	5'Phosphor
5C_chr21-BamHI_REV_1609	TCCCTCTTGGCTTGGTGCAGTTCTTGGGTATCCCTTTAGTGAGGGTTAATA	5'Phosphor
5C_chr21-BamHI_REV_1611	TCCTTTTACCTCCCCGTCCTGAGCTCGACCTCCCTTTAGTGAGGGTTAATA	5'Phosphor
5C_chr21-BamHI_REV_1614	TCCAGCCTACATCTCTCAGCTACCTTTGTATCCCTTTAGTGAGGGTTAATA	5'Phosphor
5C_chr21-BamHI_REV_1616	TCCATAGTGTCTCTAACACAGGCCAGGTACTCCCTTTAGTGAGGGTTAATA	5'Phosphor
5C_chr21-BamHI_REV_2068	TCCTCTGAAGGCATTAGGGAAGGCTCTCAGTCCCTTTAGTGAGGGTTAATA	5'Phosphor
5C_chr21-BamHI_REV_2071	TCCACTAAAGGGTATTTCCGAAGTTCTGCTTCCCTTTAGTGAGGGTTAATA	5'Phosphor
5C_chr21-BamHI_REV_2074	TCCACAAGTGGTTTTTTAGAGACAGGGTCTTCCCTTTAGTGAGGGTTAATA	5'Phosphor
5C_chr21-BamHI_REV_2237	TCCAATAGGTCTGACCCCGGCCGGTAGAGTTCCCTTTAGTGAGGGTTAATA	5'Phosphor
5C_chr21-BamHI_REV_2239	TCCATCTGGGTTTAAGATGGAATGAGCTGGTCCCTTTAGTGAGGGTTAATA	5'Phosphor
5C_chr21-BamHI_REV_2242	TCCTCTTTCTAAGGAAGTAAGAATTGGGCCTCCCTTTAGTGAGGGTTAATA	5'Phosphor
5C_chr21-BamHI_REV_2244	TCCACAAGGCCAGGGAGGTACTTGCGAATGTCCCTTTAGTGAGGGTTAATA	5'Phosphor
5C_chr21-BamHI_REV_2271	TCCCTTCTCCTGGGGACTGGCACCTTCTGATCCCTTTAGTGAGGGTTAATA	5'Phosphor
5C_chr21-BamHI_REV_2273	TCCTCCCACCTCAGGCTTCTGAGTAGGACCTCCCTTTAGTGAGGGTTAATA	5'Phosphor
5C_chr21-BamHI_REV_2276	TCCCAAGAAAGAAGCCTTGCTCTCAAATTCTCCCTTTAGTGAGGGTTAATA	5'Phosphor
5C_chr21-BamHI_REV_2277	TCCCTTTCTTCTGCTCTTTTGAGTATAGGCTCCCTTTAGTGAGGGTTAATA	5'Phosphor
5C_chr21-BamHI_REV_2279	TCCAACAGGAACCTTAAACTAAAGCTCTGATCCCTTTAGTGAGGGTTAATA	5'Phosphor
5C_chr21-BamHI_REV_2283	TCCCTTCGTGGCCCCTAGGAGATGCTGTCCTCCCTTTAGTGAGGGTTAATA	5'Phosphor
5C_chr21-BamHI_REV_2285	TCCCTAGGATGGGGACACCAGTCGAGAAGTTCCCTTTAGTGAGGGTTAATA	5'Phosphor
5C_chr21-BamHI_REV_2288	TCCCAACCCCACAGAGCCCAGGTTCGATTCTCCCTTTAGTGAGGGTTAATA	5'Phosphor
5C_chr21-BamHI_REV_2300	TCCACTAACTTCAGACTCAAGTTGTCAGCATCCCTTTAGTGAGGGTTAATA	5'Phosphor

5C_chr21-BamHI_REV_2302	TCCCAACAATGAAGCCATTTGAAACGCTGATCCCTTTAGTGAGGGTTAATA	5'Phosphor
5C_chr21-BamHI_REV_2308	TCCCTGTTTCTCAGGGTACACCTTGAGGAGTCCCTTTAGTGAGGGTTAATA	5'Phosphor
5C_chr21-BamHI_REV_2310	TCCTGAAGCAAGTTACTAAGGAAGAGACTGTCCCTTTAGTGAGGGTTAATA	5'Phosphor
5C_chr21-BamHI_REV_2313	TCCAAATCAGAGGTGTTAATGGAAGGCAACTCCCTTTAGTGAGGGTTAATA	5'Phosphor
5C_chr21-BamHI_REV_2315	TCCTGTGGGTTTCCTTCTTCCCATGGCGTGTCCCTTTAGTGAGGGTTAATA	5'Phosphor
5C_chr21-BamHI_REV_2320	TCCGGGTCTCAGGGTGGAAACTGAACCCGTTCCCTTTAGTGAGGGTTAATA	5'Phosphor
5C_chr21-BamHI_REV_2323	TCCAGTTTTAGCTTTCTTCATATGGCTAGCTCCCTTTAGTGAGGGTTAATA	5'Phosphor
5C_chr21-BamHI_REV_2423	TCCACTTCTTGAATTCAAATAGACTAGACTTCCCTTTAGTGAGGGTTAATA	5'Phosphor
5C_chr21-BamHI_REV_2425	TCCCACCCCAGGAGCTACTTCTGGAGAAGATCCCTTTAGTGAGGGTTAATA	5'Phosphor
5C_chr21-BamHI_REV_2442	TCCTTGTATGCAGAGCAGCACCTATAATAGTCCCTTTAGTGAGGGTTAATA	5'Phosphor
5C_chr21-BamHI_REV_2444	TCCTAGGGTGCAGAAAAATTCTGATGTGCATCCCTTTAGTGAGGGTTAATA	5'Phosphor
5C_chr21-BamHI_REV_2445	TCCCTTGCATGTGCATGTCTATGCGAGAGATCCCTTTAGTGAGGGTTAATA	5'Phosphor
5C_chr21-BamHI_REV_2448	TCCTTCCTGACTTAATTGGCACTTGACCTATCCCTTTAGTGAGGGTTAATA	5'Phosphor
5C_chr21-BamHI_REV_2459	TCCAGGCACAAGTTGTCAGGACACAGCTTCTCCCTTTAGTGAGGGTTAATA	5'Phosphor
5C_chr21-BamHI_REV_2461	TCCTGTATCAGATCATTCGGGAGCTGTCAGTCCCTTTAGTGAGGGTTAATA	5'Phosphor
5C_chr21-BamHI_REV_2493	TCCCATTACTTCCCAATCAATTCCGTAACTTCCCTTTAGTGAGGGTTAATA	5'Phosphor
5C_chr21-BamHI_REV_2495	TCCCAAGGCAGATTGGCAGACCGTAGGAGTTCCCTTTAGTGAGGGTTAATA	5'Phosphor
5C_chr21-BamHI_REV_2520	TCCACAGGTACATCTGTTGTACTAGGTGCATCCCTTTAGTGAGGGTTAATA	5'Phosphor
5C_chr21-BamHI_REV_2522	TCCTCAATTCTTGCCTTCTCAGAAGAAAGATCCCTTTAGTGAGGGTTAATA	5'Phosphor
5C_chr21-BamHI_REV_2612	TCCTCCTATGGATTTCCTTGTTCTGCTCCTTCCCTTTAGTGAGGGTTAATA	5'Phosphor
5C_chr21-BamHI_REV_2615	TCCTTGCTATTTGTCTCCTTGCCATTTTGGTCCCTTTAGTGAGGGTTAATA	5'Phosphor
5C_chr21-BamHI_REV_2619	TCCTTTGTGGCTTCTCAGCTGTGTTACACTTCCCTTTAGTGAGGGTTAATA	5'Phosphor
5C_chr21-BamHI_REV_2667	TCCCCTTCCCAATCAAAGAGAGGACTTAGGTCCCTTTAGTGAGGGTTAATA	5'Phosphor
5C_chr21-BamHI_REV_2669	TCCCACTTCTGCAACAACTAACCCACTCGGTCCCTTTAGTGAGGGTTAATA	5'Phosphor
5C_chr21-BamHI_REV_2670	TCCTTGGGGTTCTGGGGCTTGGTCGTTTACTCCCTTTAGTGAGGGTTAATA	5'Phosphor
5C_chr21-BamHI_REV_2672	TCCAGGTTTAGAGGCCCCTGCCACGCAGTATCCCTTTAGTGAGGGTTAATA	5'Phosphor
5C_chr21-BamHI_REV_2737	TCCCTTGAGCCCTTTAGCCAGGAGAAACTCTCCCTTTAGTGAGGGTTAATA	5'Phosphor

5C_chr21-BamHI_REV_2739	TCCTCATCTCTCACCTTATACAAATATCAATCCCTTTAGTGAGGGTTAATA	5'Phosphor
5C_chr21-BamHI_REV_2742	TCCCCGTGGCCTCTCAGTGTGCGCGCCGTCTCCCTTTAGTGAGGGTTAATA	5'Phosphor
5C_chr21-BamHI_REV_2744	TCCTCACAGCCCTCACTTGCAGGCTGAACGTCCCTTTAGTGAGGGTTAATA	5'Phosphor
5C_chr21-BamHI_REV_2778	TCCAGTTTCAGCTTTCTATATATGGCTAGCTCCCTTTAGTGAGGGTTAATA	5'Phosphor
5C_chr21-BamHI_REV_2780	TCCACTGCTTTGGCCTCCCAAAGGCGACCCTCCCTTTAGTGAGGGTTAATA	5'Phosphor
5C_chr21-BamHI_REV_2820	TCCAGGCTAAATCGTTCCAATCTCCATAGATCCCTTTAGTGAGGGTTAATA	5'Phosphor
5C_chr21-BamHI_REV_2822	TCCTAGAAGAGAGATAGGGAGGAGGAGAAGTCCCTTTAGTGAGGGTTAATA	5'Phosphor
5C_chr21-BamHI_REV_2869	TCCAGGCCAGGAATCTGAAACGTCAGATAATCCCTTTAGTGAGGGTTAATA	5'Phosphor
5C_chr21-BamHI_FOR_2870	TCCTCCCGCCTTGGCCTCCTGTCATCGACCTCCCTTTAGTGAGGGTTAATA	5'Phosphor
5C_chr21-BamHI_REV_2871	TCCCTTACCTCTTCCTTAAGCTGATAGTTGTCCCTTTAGTGAGGGTTAATA	5'Phosphor
5C_chr21-BamHI_REV_2876	TCCATCTAAAATGGTTGCTGTCTAACAGCTTCCCTTTAGTGAGGGTTAATA	5'Phosphor
5C_chr21-BamHI_REV_2878	TCCGGGCTGTAGGTTAGGATCTGGCTGTTCTCCCTTTAGTGAGGGTTAATA	5'Phosphor
5C_chr21-BamHI_REV_3064	TCCTCTCTCTTCACAAAGCAGACATAGGCATCCCTTTAGTGAGGGTTAATA	5'Phosphor
5C_chr21-BamHI_REV_3066	TCCTGTTAGCCTTTCTCCCATCCTTTCCGTTCCCTTTAGTGAGGGTTAATA	5'Phosphor
5C_chr21-BamHI_REV_3077	TCCCAACTCTTTTGAGTGAGGAGTAAGGAGTCCCTTTAGTGAGGGTTAATA	5'Phosphor
5C_chr21-BamHI_REV_3081	TCCCATCAGCTGCCGCAGCAAGTTTCGGTCTCCCTTTAGTGAGGGTTAATA	5'Phosphor
5C_chr21-BamHI_REV_3084	TCCTAGCCTCCAGAACTGTGGGAAGATGTTTCCCTTTAGTGAGGGTTAATA	5'Phosphor
5C_chr21-BamHI_REV_3100	TCCTCCCAGAGCCTCCGAGGCACGACGGATTCCCTTTAGTGAGGGTTAATA	5'Phosphor
5C_chr21-BamHI_REV_3102	TCCTTCATGAATGGCTTAGCACCATCACCATCCCTTTAGTGAGGGTTAATA	5'Phosphor
5C_chr21-BamHI_REV_3162	TCCAGCCGCCGCAGGACACAATCCTGCACATCCCTTTAGTGAGGGTTAATA	5'Phosphor
5C_chr21-BamHI_REV_3164	TCCCCACATGCAGAGCCAGGCTATGAGTAATCCCTTTAGTGAGGGTTAATA	5'Phosphor
5C_chr21-BamHI_REV_3166	TCCGAGGAGAGATGGAGGGAAGAGGTCTCCTCCCTTTAGTGAGGGTTAATA	5'Phosphor
5C_chr21-BamHI_REV_3168	TCCCCATTTTACAGAGGACAAAACTGAGGCTCCCTTTAGTGAGGGTTAATA	5'Phosphor
5C_chr21-BamHI_REV_3170	TCCGCCTGAAGGTTGGATCACGGCCAGTGTTCCCTTTAGTGAGGGTTAATA	5'Phosphor
5C_chr21-BamHI_REV_3172	TCCACCCGGGGGGCTACAAAAGGGCCCGAATCCCTTTAGTGAGGGTTAATA	5'Phosphor
5C_chr21-BamHI_REV_3175	TCCAGGCTCACCATGGGCCCGGCTCCCGGTTCCCTTTAGTGAGGGTTAATA	5'Phosphor
5C_chr21-BamHI_REV_3202	TCCTGGGGGCGGTTCCCTCAACGACAAGAGTCCCTTTAGTGAGGGTTAATA	5'Phosphor

5C_chr21-BamHI_REV_3205	TCCCTGGACCAGCAACAGCAGGTAGCGGGGTCCCTTTAGTGAGGGTTAATA	5'Phosphor
5C_chr21-BamHI_REV_3227	TCCTTCCCAGCAAGCCAGGAACACAAAGAGTCCCTTTAGTGAGGGTTAATA	5'Phosphor
5C_chr21-BamHI_REV_3230	TCCCCGGGCCTCCTTAGGCATTACAATAGATCCCTTTAGTGAGGGTTAATA	5'Phosphor
5C_chr21-BamHI_REV_3233	TCCACTCAGAGCTGGCAGCCCGTCCCCCTCTCCCTTTAGTGAGGGTTAATA	5'Phosphor
5C_chr21-BamHI_REV_3236	TCCAGCCCTACACACAGGCAAACTCCAGCGTCCCTTTAGTGAGGGTTAATA	5'Phosphor
5C_chr21-BamHI_REV_3239	TCCTTTCTGCCAGCTCACCACCGGTCTACCTCCCTTTAGTGAGGGTTAATA	5'Phosphor
5C_chr21-BamHI_REV_3242	TCCCTGCAAGTCTCGGGCAATCCCCCCACCTCCCTTTAGTGAGGGTTAATA	5'Phosphor
5C_chr21-BamHI_REV_3349	TCCTCCTACCTTGGCCTCCCAAAATGTACCTCCCTTTAGTGAGGGTTAATA	5'Phosphor
5C_chr21-BamHI_REV_3351	TCCAAGGAGTAATCTTGACTTTCAAGTCTTTCCCTTTAGTGAGGGTTAATA	5'Phosphor
5C_chr21-BamHI_REV_3354	TCCAGAAGGACAAACACTGTACGATTCCGGTCCCTTTAGTGAGGGTTAATA	5'Phosphor
5C_chr21-BamHI_REV_3356	TCCCGCCATGCTTCATTTAGAGGAACCACCTCCCTTTAGTGAGGGTTAATA	5'Phosphor
5C_chr21-BamHI_REV_3399	TCCAGCCCGGGGAGCCAGTGGGACCCAAGATCCCTTTAGTGAGGGTTAATA	5'Phosphor
5C_chr21-BamHI_REV_3403	TCCAGACCTGGAAGGAAATGGGGTCTGTGTTCCCTTTAGTGAGGGTTAATA	5'Phosphor
5C_chr21-BamHI_REV_3405	TCCATAGCTCCCAGCCACCTGGCCCACCGTTCCCTTTAGTGAGGGTTAATA	5'Phosphor
5C_chr21-BamHI_REV_3407	TCCATCTAGCTTCCTGCTTCTCAAAGAACATCCCTTTAGTGAGGGTTAATA	5'Phosphor
5C_chr21-BamHI_REV_3455	TCCTAGAGCAAGTGGCAGGAGACCAATCACTCCCTTTAGTGAGGGTTAATA	5'Phosphor
5C_chr21-BamHI_REV_3457	TCCTTTTCCTTCTGAAACAAATGTTCTTTTTCCCTTTAGTGAGGGTTAATA	5'Phosphor
5C_chr21-BamHI_REV_3467	TCCAAAGAACAGGTGAAAAATTTGTTTCGGTCCCTTTAGTGAGGGTTAATA	5'Phosphor
5C_chr21-BamHI_REV_3469	TCCCGAGTGAGCCAAAGAAGGAAACAGAAATCCCTTTAGTGAGGGTTAATA	5'Phosphor
5C_chr21-BamHI_REV_3471	TCCACCCTCTTCAGCCTCCTGAGTAGGATCTCCCTTTAGTGAGGGTTAATA	5'Phosphor
5C_chr21-BamHI_REV_3552	TCCGCTGCACCCCTTGGCTCTCCGGAGAGATCCCTTTAGTGAGGGTTAATA	5'Phosphor
5C_chr21-BamHI_REV_3554	TCCAGGGCCTCCTTCCTTGCAATATGAGGGTCCCTTTAGTGAGGGTTAATA	5'Phosphor
5C_chr21-BamHI_REV_3567	TCCTCAGAGCCAGTGTTAAGCAAAGAGGGATCCCTTTAGTGAGGGTTAATA	5'Phosphor
5C_chr21-BamHI_REV_3570	TCCACCTGAGCCGTGATCCTGGCCAGTGGATCCCTTTAGTGAGGGTTAATA	5'Phosphor
5C_chr21-BamHI_REV_3723	TCCTTCTAGCAAATCCCCCTCTCTGCTTCGTCCCTTTAGTGAGGGTTAATA	5'Phosphor
5C_chr21-BamHI_REV_3725	TCCTGAGTCCTAGGGTGAGATTCCGAACAATCCCTTTAGTGAGGGTTAATA	5'Phosphor
5C_chr21-BamHI_REV_3783	TCCAAGTTAAGCTGGTGCAGGTGAGGTCCGTCCCTTTAGTGAGGGTTAATA	5'Phosphor

5C_chr21-BamHI_REV_3785	TCCATCACCACACAGCAGGGGGTTTTAGGGTCCCTTTAGTGAGGGTTAATA	5'Phosphor
5C_chr21-BamHI_REV_3787	TCCATGTCATGACACACTTGTCAAAACCCATCCCTTTAGTGAGGGTTAATA	5'Phosphor
5C_chr21-BamHI_REV_3789	TCCCATCACTCCTTCTGCGTTTACTCGGACTCCCTTTAGTGAGGGTTAATA	5'Phosphor
5C_chr21-BamHI_REV_3803	TCCTAACTCAATCCCATCCCCAAAGACACATCCCTTTAGTGAGGGTTAATA	5'Phosphor
5C_chr21-BamHI_REV_3805	TCCAACCTGACAATCCACTGAACCTCAGAATCCCTTTAGTGAGGGTTAATA	5'Phosphor
5C_chr21-BamHI_REV_3806	TCCCCTGGCACTCCCGGGGCGGTTTCTTGGTCCCTTTAGTGAGGGTTAATA	5'Phosphor
5C_chr21-BamHI_REV_3809	TCCATGGACGCTTCCTTCTTTCTGGCACCTTCCCTTTAGTGAGGGTTAATA	5'Phosphor
5C_chr21-BamHI_REV_3915	TCCCCAATTTCCCAAAGTCATATACAGAATTCCCTTTAGTGAGGGTTAATA	5'Phosphor
5C_chr21-BamHI_REV_3917	TCCTGGGTGCCAGAGGCTGGCCCTCCCTCCTCCCTTTAGTGAGGGTTAATA	5'Phosphor
5C_chr21-BamHI_REV_3920	TCCTGAGCTAAGGGAACACTTTAGTTCATTTCCCTTTAGTGAGGGTTAATA	5'Phosphor
5C_chr21-BamHI_REV_3922	TCCCACCTCGCCCTCAAATGTGTGAGGATATCCCTTTAGTGAGGGTTAATA	5'Phosphor
5C_chr21-BamHI_REV_3996	TCCTCTGTCCCGGGGCGATCTAGGAGGTCTTCCCTTTAGTGAGGGTTAATA	5'Phosphor
5C_chr21-BamHI_REV_3998	TCCAGCCCCAGTTAGGAACAGGGGCCCTCATCCCTTTAGTGAGGGTTAATA	5'Phosphor
**Chr16 Forward Oligos ID**	**Sequences (5'-3')**	
5C_chr16-BamHI_FOR_2	TAATACGACTCACTATAGCCTTAAGGTTCTGCCCTCTCTGGGGGTCTGGA	
5C_chr16-BamHI_FOR_3	TAATACGACTCACTATAGCCGGCTCTACCTATAATCTCAGCATTTTGGGA	
5C_chr16-BamHI_FOR_5	TAATACGACTCACTATAGCCTTTTCCTAAAGAGAAACCCATGAGGAGGGA	
5C_chr16-BamHI_FOR_9	TAATACGACTCACTATAGCCTCCACCCCTTCACTCGTATTTTCTCTTGGA	
5C_chr16-BamHI_FOR_11	TAATACGACTCACTATAGCCCCGCATTACACCGGCAGCAAACCCCTTGGA	
5C_chr16-BamHI_FOR_13	TAATACGACTCACTATAGCCTCGGACTGATTCCTGGTTGGGGGTTGGGGA	
5C_chr16-BamHI_FOR_15	TAATACGACTCACTATAGCCAAAAAAAAAAAAAAAACCCACAAAACTGGA	
5C_chr16-BamHI_FOR_17	TAATACGACTCACTATAGCCAACCTTCCATGTGCGGATGATTGAGATGGA	
5C_chr16-BamHI_FOR_19	TAATACGACTCACTATAGCCCCCTATAGTGGCAGCAGACAAACCCTTGGA	
5C_chr16-BamHI_FOR_21	TAATACGACTCACTATAGCCCAATAGTATTCGAAGTATTCTACAATAGGA	
5C_chr16-BamHI_FOR_24	TAATACGACTCACTATAGCCCTGTGTATCCAAAAATACTTAAAAGCAGGA	
5C_chr16-BamHI_FOR_28	TAATACGACTCACTATAGCCACTTCCTCAACGTGGGACAAGAACTTTGGA	

5C_chr16-BamHI_FOR_30	TAATACGACTCACTATAGCCATGTGTTCACCAAGCTGGAAGATCTCTGGA	
5C_chr16-BamHI_FOR_32	TAATACGACTCACTATAGCCATTGACAATATTTTGTCTAAACATGTGGGA	
5C_chr16-BamHI_FOR_34	TAATACGACTCACTATAGCCAATGTTAAAATATGTATACTTACACGTGGA	
5C_chr16-BamHI_FOR_36	TAATACGACTCACTATAGCCGATACTCTCCTAAACAGAAAGCAACGGGGA	
5C_chr16-BamHI_FOR_38	TAATACGACTCACTATAGCCTGAACAGAAGGATGACACATGAAGGTTGGA	
5C_chr16-BamHI_FOR_40	TAATACGACTCACTATAGCCTGGACAGAAGTTCTTTTGTAGAACGAGGGA	
5C_chr16-BamHI_FOR_42	TAATACGACTCACTATAGCCAAAGGTGCTGGAAGAGGGACAGCCAAAGGA	
5C_chr16-BamHI_FOR_44	TAATACGACTCACTATAGCCTCTTGCTATGATAGAAAATTGCTTTTGGGA	
5C_chr16-BamHI_FOR_46	TAATACGACTCACTATAGCCCGTGAGTTGCAGAGAAGCAAAAGGACTGGA	
**Chr16 Reverse Oligos ID**	**Sequences (5'-3')**	
5C_chr16-BamHI_REV_1	TCCAGCTTGGTCTCCACTCCTGACGTGACGTCCCTTTAGTGAGGGTTAATA	5'phosphor
5C_chr16-BamHI_REV_4	TCCATGATAACAGATACAGGCCCTGATGCATCCCTTTAGTGAGGGTTAATA	5'phosphor
5C_chr16-BamHI_REV_6	TCCTACATTGCACCTAGTTGCCACGCGAGGTCCCTTTAGTGAGGGTTAATA	5'phosphor
5C_chr16-BamHI_REV_10	TCCTTTAGGTAGTCCTTTTGTCATGAACACTCCCTTTAGTGAGGGTTAATA	5'phosphor
5C_chr16-BamHI_REV_12	TCCAGGGAGATATTGGAGGTAAGCAAATATTCCCTTTAGTGAGGGTTAATA	5'phosphor
5C_chr16-BamHI_REV_14	TCCTTATATTATTAAGAAAATTAAGATCAGTCCCTTTAGTGAGGGTTAATA	5'phosphor
5C_chr16-BamHI_REV_16	TCCAGTGTACAACATGGTGACTGTAGTTAATCCCTTTAGTGAGGGTTAATA	5'phosphor
5C_chr16-BamHI_REV_18	TCCTAACAAAAAATGTGTTTTATAATTCACTCCCTTTAGTGAGGGTTAATA	5'phosphor
5C_chr16-BamHI_REV_20	TCCAGTATCTCCACTATTCCAGTGGGATTTTCCCTTTAGTGAGGGTTAATA	5'phosphor
5C_chr16-BamHI_REV_23	TCCAACTTTATTTTTTGCCATTTGTTCATATCCCTTTAGTGAGGGTTAATA	5'phosphor
5C_chr16-BamHI_REV_25	TCCCATTCCTGCTATAGTTAATATTTTCTGTCCCTTTAGTGAGGGTTAATA	5'phosphor
5C_chr16-BamHI_FOR_26	TCCCTACCTTCCACCATATACAAAAATTAATCCCTTTAGTGAGGGTTAATA	5'phosphor
5C_chr16-BamHI_REV_27	TCCAAGGCTGATTTAAGAAACACAAATCAATCCCTTTAGTGAGGGTTAATA	5'phosphor
5C_chr16-BamHI_REV_29	TCCTTAGGATGTCCCTTTGCCAGCTGCTTTTCCCTTTAGTGAGGGTTAATA	5'phosphor
5C_chr16-BamHI_REV_31	TCCCTTCCTTACACCTTATACAAAAATTAATCCCTTTAGTGAGGGTTAATA	5'phosphor
5C_chr16-BamHI_REV_33	TCCCAGGCTTGGTCAATTTGACTTTCATGTTCCCTTTAGTGAGGGTTAATA	5'phosphor

5C_chr16-BamHI_REV_35	TCCTCTAAAGTCGAGATGAATATGCTAATTTCCCTTTAGTGAGGGTTAATA	5'phosphor
5C_chr16-BamHI_REV_37	TCCTACCCTGCAACACCTTGATTTTGGACGTCCCTTTAGTGAGGGTTAATA	5'phosphor
5C_chr16-BamHI_REV_39	TCCTTAAAGTAGAACTACACCGGTGTGAGTTCCCTTTAGTGAGGGTTAATA	5'phosphor
5C_chr16-BamHI_REV_41	TCCGGTGAGGTCCTTTAACCCCAACTTTACTCCCTTTAGTGAGGGTTAATA	5'phosphor
5C_chr16-BamHI_REV_43	TCCTTTTGGTTAGAAGGTCAAAGAAATAGTTCCCTTTAGTGAGGGTTAATA	5'phosphor
5C_chr16-BamHI_REV_45	TCCCTGTTTCATGGAATAGGATATATCTATTCCCTTTAGTGAGGGTTAATA	5'phosphor
