# Supplementary material for: Ligand dependent gene regulation by transient ERα clustered enhancers
Source: PLoS Genet. 2020 Jan 6;16(1):e1008516. doi: 10.1371/journal.pgen.1008516 (PMC6975561; doi:10.1371/journal.pgen.1008516)
Supplement: S2 Table — (PDF) [file pgen.1008516.s014.pdf]

TableS2: 5C reads and Percent Alignment:

Sample	Total Reads	Aligned Reads	Percent Alignment	Total Contacts	All Possible Contacts
ICI 5C Rep1	25612149	25320119	0.988597989	17839	23095
ICI 5C Rep2	25491622	25325094	0.993467344	17882	23095
E2 5C Rep1	26623491	26140530	0.981859592	18206	23095
E2 5C Rep2	26285590	26007082	0.989404537	18060	23095
