# Supplementary material for: Ligand dependent gene regulation by transient ERα clustered enhancers
Source: PLoS Genet. 2020 Jan 6;16(1):e1008516. doi: 10.1371/journal.pgen.1008516 (PMC6975561; doi:10.1371/journal.pgen.1008516)
Supplement: S3 Table — (PDF) [file pgen.1008516.s015.pdf]

TableS3: Sequences of gRNA and primers:
Primers used for ChIP-qPCRs:

GREB1 LDEC	Forward Primer (5' to 3')	Reverse Primer (5' to 3')
GREB1_peak1	AATGGGAGTGATCTGAGTGGTT	TTTCATGTATGAGGCAATGGTC
GREB1_peak2	CTCCTGGCAGAGCAAAACAT	CAGGCAAAGAAATGAACCTTG
GREB1_peak3	GGGAGTCTGAGTTCCTATGCAG	CTGAAATCAGTCAGCAGTTTCG
GREB1_peak4	TTTCCTGGAGGGACACTCTTTA	AATGAGCTGGGTCATGGTAACT
GREB1_peak5	ACACCAAATATTGTTGGGCTTC	CACCCACTAGGAATCTTGGAAC
GREB1_peak6	ACAGGAGCTCTTTGCTCAAATC	AGCCATATCCGCCTAAGTAAAA
GREB1_peak7	CTGGAAGCCCTGTGAGATGT	ATGCTGTCACCTCCTGTTCC
GREB1_peak8	GCCCAGGAGACAGGTTGTAA	TCCTGCAGAGGTGGCTATTT

TFF1 LDEC	Forward Primer (5' to 3')	Reverse Primer (5' to 3')
TFF1_Peak1	TCACTCTGCACAGAACTTCCTC	CCTTCCTGTTTTACACGTGGTG
TFF1_Peak2	CTGGACAGAGAGTTGGGTCAT	GAGGGGACTTTTCCATGCTATC
TFF1_Peak3	CCTCCTCTCTGCTCCAAAGG	GACCTCACCACATGTCGTCTC
TFF1_Peak4	GCAGCCAGGAAAAGGAGTGA	ACGTGTACGGTGGCATCATC
TFF1_Peak4.2	GCCCAGGACTAGCTGTGATCT	GTGTCACCTCCTTCCTGGACT
TFF1_Peak5	ACCACCAGGAGCTAGGAAGAG	GGATGCTACTTCCCCTCCAT
TFF1_Peak6	ATCCTCTCTCCACCCTCACAC	GGGAACTGACACAGCCTTTC
TFF1_Peak7	CAGAGGCTCAGTCAAGGTCAC	AGGGGCTCTCAGGTCAGTCT

gRNAs used for persistent site deletion/blocking:

TFF1_gRNA1	CTGGCAACGACCTGTCCCAA
TFF1_gRNA2	ACAACCACTGGGTCACGCCC
GREB1_gRNA1	ACAAGGTCAGCTCCACCCGT
GREB1_gRNA2	CTAGTCCTTTGTTGTATATG

Surveyor Oligos:

TFF1 F	CCTCCTTCTTCCTCCTCCAC
TFF1 R	GATCTCGAGATTCCTGGGCTTGTAGCTGG
GREB1 F	CTTCATGTCTTTCGCCCATT
GREB1 R	GCCTACCACAAGGTCAGCTC

Primers for qRT-PCRs:

mRNA	Forward Primer (5'-3')	Reverse Primer (5'-3')
TFF1 mRNA	CCATGGAGAACAAGGTGATCTGCGCCCT	GCAGCCCTTATTTGCACACTGGGA
TFF2 mRNA	CGAACTGCGGCTTCCCTGGAATCACCAG	GCCCGGGTAGCCACAGTTTCTTCGGTC
TFF3 mRNA	GCTGCTTTGACTCCAGGATCCCTGGAGTG	TGCCTGGCAGCAATCACAGCCGGGCAA
NRIP1 mRNA	CGGCCTGGGGAAGTGTTTGGATTGTGAGC	CAGTGTTCGTCTGTCTCCAAGCTCTGAGC
ESR1 mRNA	TGTGTC CAG CCACCA ACC AG	TTCAAC ATT CTC CCTCCTCTTCGG
FOXA1 mRNA	GGGTGG CTC CAG GAT GTTAGG	GGG TCA TGT TGC CGC TCG TAG
GREB1 mRNA	TACCTGGTCCGTAATGCACA	GACCCATTGCTGCGTTTAGT
GAPDH mRNA	CGCTCTCTGCTCCTCCTGTT	CCATGGTGTCTGAGCGATGT
