# Supplementary material for: Ligand dependent gene regulation by transient ERα clustered enhancers
Source: PLoS Genet. 2020 Jan 6;16(1):e1008516. doi: 10.1371/journal.pgen.1008516 (PMC6975561; doi:10.1371/journal.pgen.1008516)
Supplement: S4 Table — (DOCX) [file pgen.1008516.s016.docx]

**TableS4: Accession Numbers of NGS datasets:**

| **Accession Number** | **Experiment** | **Reference** |
| --- | --- | --- |
| GSM1115990 | ERa_ChIPSeq_repeat1 | Liu Z. et al., 2014 |
| GSM1115991 | ERa_ChIPSeq_repeat2 | Liu Z. et al., 2014 |
| GSM1115992 | H3K27ac_ChIPSeq_E2 | Li W, Notani D. et al., 2013 |
| GSM1115993 | H3K27ac_ChIPSeq_EtOH | Li W, Notani D. et al., 2013 |
| GSM1295590 | siCTL_ChIP-Seq | Caizzi et al., 2012 |
| GSM1295591 | siERα_ChIP-Seq | Caizzi et al., 2012 |
| GSM1115995 | GROSeq_E2_repeat1 | Li W, Notani D. et al., 2013 |
| GSM1115996 | GROSeq_E2_repeat2 | Li W, Notani D. et al., 2013 |
| GSM1115997 | GROSeq_EtOH_repeat1 | Li W, Notani D. et al., 2013 |
| GSM1115998 | GROSeq_EtOH_repeat2 | Li W, Notani D. et al., 2013 |
| GSM678535 | GRO-seq_Vehicle_rep1 | Hah N. et al., 2011 |
| GSM678536 | GRO-seq_Vehicle_rep2 | Hah N. et al., 2011 |
| GSM678537 | GRO-seq_E2_10m_rep1 | Hah N. et al., 2011 |
| GSM678538 | GRO-seq_E2_10m_rep2 | Hah N. et al., 2011 |
| GSM678539 | GRO-seq_E2_40m_rep1 | Hah N. et al., 2011 |
| GSM678540 | GRO-seq_E2_40m_rep2 | Hah N. et al., 2011 |
| GSM678541 | GRO-seq_E2_160m_rep1 | Hah N. et al., 2011 |
| GSM678542 | GRO-seq_E2_160m_rep2 | Hah N. et al., 2011 |
| GSM822389 | MCF-7 vehicle DNaseI | He HH. et al., 2012 |
| GSM822390 | MCF-7 E2 DNaseI | He HH. et al., 2012 |
| GSM1469999 | Med1_ChIPseq_Veh_Exp9 | Liu Z. et al., 2014 |
| GSM1470000 | Med1_ChIPseq_E2_Exp9 | Liu Z. et al., 2014 |
| GSM1470025 | FoxA1_ChIPseq_Veh_Exp18 | Liu Z. et al., 2014 |
| GSM1470026 | FoxA1_ChIPseq_E2_Exp18 | Liu Z. et al., 2014 |
| GSM1470023 | ERa_ChIPseq_siCTL_E2_Exp17 | Liu Z. et al., 2014 |
| GSM1470024 | ERa_ChIPseq_siFoxA1_E2_Exp17 | Liu Z. et al., 2014 |
| GSM970212 | GIS-Ruan_ChiaPet_MCF-7_ERalpha_a | PRJNA63443 Production ENCODE epigenomic data |
| GSM2467220 | MCF7_NoTreat_ERalpha | Dzida T. et al., 2017 |
| GSM2467221 | MCF7_E2_5min_ERalpha | Dzida T. et al., 2017 |
| GSM2467222 | MCF7_E2_10min_ERalpha | Dzida T. et al., 2017 |
| GSM2467223 | MCF7_E2_20min_ERalpha | Dzida T. et al., 2017 |
| GSM2467224 | MCF7_E2_40min_ERalpha | Dzida T. et al., 2017 |
| GSM2467225 | MCF7_E2_80min_ERalpha | Dzida T. et al., 2017 |
| GSM2467226 | MCF7_E2_160min_ERalpha | Dzida T. et al., 2017 |
| GSM2467227 | MCF7_E2_320min_ERalpha | Dzida T. et al., 2017 |
| GSM2467228 | MCF7_E2_640min_ERalpha | Dzida T. et al., 2017 |
| GSM2467229 | MCF7_E2_1280_min_ERalpha | Dzida T. et al., 2017 |
| GSM3436593 | MCF7_0nM_R1: 3e Hi-C human 0 nM MCF7 cell rep.1 | Rodriguez et al., 2018 |
| GSM3436594 | MCF7_0nM_R2: 3e Hi-C human 0 nM MCF7 cell rep.2 | Rodriguez et al., 2018 |
| GSM3436597 | MCF7_SatE2_R1: 3e Hi-C human Saturated E2 MCF7 cell rep.1 | Rodriguez et al., 2018 |
| GSM3436598 | MCF7_SatE2_R2: 3e Hi-C human Saturated E2 MCF7 cell rep.2 | Rodriguez et al., 2018 |
| GSM4044331 | ERα ChIP-seq WT MCF7 E2 | GSE136302 (This study) |
| GSM4044330 | ERα ChIP-seq WT MCF7 7 days stripping | GSE136302 (This study) |
| GSM4044324 | ERα ChIP-seq ΔTFF1_PS MCF7 E2 rep1 | GSE136302 (This study) |
| GSM4044325 | ERα ChIP-seq ΔTFF1_PS MCF7 E2 rep2 | GSE136302 (This study) |
| GSM4044326 | 5C_MCF7_ICI rep1 | GSE136302 (This study) |
| GSM4044327 | 5C_MCF7_ICI rep2 | GSE136302 (This study) |
| GSM4044328 | 5C_MCF7_E2 rep1 | GSE136302 (This study) |
| GSM4044329 | 5C_MCF7_E2 rep2 | GSE136302 (This study) |
